# Lactic acid production from food waste at an anaerobic digestion biorefinery: effect of digestate recirculation and sucrose supplementation

**DOI:** 10.3389/fbioe.2023.1177739

**Published:** 2023-05-12

**Authors:** Christopher H. Bühlmann, Bede S. Mickan, Stephan Tait, Damien J. Batstone, Parisa A. Bahri

**Affiliations:** ^1^ Discipline of Engineering and Energy, Murdoch University, Perth, WA, Australia; ^2^ School of Agriculture and Environment, The University of Western Australia, Perth, WA, Australia; ^3^ Institute of Agriculture, The University of Western Australia, Perth, WA, Australia; ^4^ Richgro Garden Products, Jandakot, WA, Australia; ^5^ Centre for Agricultural Engineering, University of Southern Queensland, Toowoomba, QLD, Australia; ^6^ Australian Centre for Water and Environmental Biotechnology, The University of Queensland, St Lucia, Brisbane, QLD, Australia

**Keywords:** fermentation, lactic acid, food waste, nitrogen, digestate, mixed culture, organic acid

## Abstract

Low lactic acid (LA) yields from direct food waste (FW) fermentation restrict this production pathway. However, nitrogen and other nutrients within FW digestate, in combination with sucrose supplementation, may enhance LA production and improve feasibility of fermentation. Therefore, this work aimed to improve LA fermentation from FWs by supplementing nitrogen (0–400 mgN·L^−1^) as NH_4_Cl or digestate and dosing sucrose (0–150 g·L^−1^) as a low-cost carbohydrate. Overall, NH_4_Cl and digestate led to similar improvements in the rate of LA formation (0.03 ± 0.02 and 0.04 ± 0.02 h^−1^ for NH_4_Cl and digestate, respectively), but NH_4_Cl also improved the final concentration, though effects varied between treatments (5.2 ± 4.6 g·L^−1^). While digestate altered the community composition and increased diversity, sucrose minimised community diversion from LA, promoted *Lactobacillus* growth at all dosages, and enhanced the final LA concentration from 25 to 30 g·L^−1^ to 59–68 g·L^−1^, depending on nitrogen dosage and source. Overall, the results highlighted the value of digestate as a nutrient source and sucrose as both community controller and means to enhance the LA concentration in future LA biorefinery concepts.

## 1 Introduction

Food waste (FW) is edible food lost along the supply and consumption chain which is produced in large quantities, and continues to increase with a growing global population (Wolka and Melaku, 2015). Recent estimates have suggested 1.6 billion tonnes of FW is produced globally each year, costing the global economy USD 2.6 trillion per annum (WBA, 2018). Consequently, there is a pressing need to develop closed-loop technologies that beneficially utilise and reduce FW to mitigate associated adverse impacts. Anaerobic digestion (AD) is a technology now widely used to process FWs into renewable biogas energy, and digestate as a fertilizer nutrient source. However, the economic feasibility of FW AD heavily relies on gate-fees, which are subject to government policy or subsidy support (Bastidas-Oyanedel and Schmidt, 2018). To address this, recent research has proposed and demonstrated pairing AD with lactic acid (LA) production and recovery, aimed at generating additional revenue via a LA-AD biorefinery concept to make FW AD facilities more economically feasible (Kim et al., 2016; Demichelis et al., 2018; Bühlmann et al., 2022). LA, which is generally produced from expensive first-generation feedstocks (e.g., corn and beet) (Malacara-Becerra et al., 2022), is a valuable commodity chemical with uses in various industries including the food, pharmaceutical, and textile industries or as a raw material for the production of biodegradable bioplastics (Kim et al., 2016). To reduce dependency on traditional feedstocks and on food resources, literature has explored LA production from FW and identified production is technically feasible at both lab (Kim et al., 2016; Pleissner et al., 2017) and commercial scale (Bühlmann et al., 2021). However, barriers which limit LA production from FWs still exist including 1) the slow hydrolysis rate of FW (Zhang et al., 2020b), 2) formation of competitor metabolite by-products, and 3) the low LA yield (Yousuf et al., 2018). Arguably, the last listed is currently one of the most significant constraints on the development of commercial LA biorefineries, as it directly limits the final LA concentration achieved, therefore elevating recovery costs (Alves de Oliveira et al., 2018).

FW fermentation to LA is thought to be limited by the inability of LA bacteria to fully utilise the FW substrate. While higher substrate concentrations promote LA production (to a point) (Pleissner et al., 2017; Yousuf et al., 2018; Pu et al., 2019) and the growth of LA bacteria (Pu et al., 2019), overall yields generally reduce with higher substrate concentrations, lowering process efficiency. Pre-treated can enhance LA production (Kwan et al., 2016; Pleissner et al., 2016), likely by increasing substrate bioavailability, but is generally costly to operate (Surendra et al., 2015). Alternatively, FWs may be supplemented with simple carbohydrates, such as sucrose (commonly used LA production Olszewska-Widdrat et al., 2020), to increase the concentration of bioavailable substrate, and hence, elevate the final LA concentration. As the FW context is highly variable (Bühlmann et al., 2021), this approach would be realistic in a FW biorefinery where it would aid in reducing process variability related to the final LA concentration while minimising downstream processing costs.

Nitrogen (N) is essential for synthesising carrier proteins, with ammonium being the preferred N source for all bacteria, including LA bacteria (Zhang et al., 2020b). Recent literature has shown NH_4_Cl can be an effective N supplement to enhance FW fermentation to LA, yielding a 2.0–2.4 fold increase in LA concentration following supplementation with 300–400 mgN·L^−1^ (Zhang et al., 2020a; Zhang et al., 2020b). However, FW AD digestate naturally contains elevated ammonium concentrations (3,280–5,000 ppm N (Serna-Maza et al., 2015; Buhlmann et al., 2018)), and so may be a promising low-cost N source for LA fermentation. Limited available research has indeed shown the benefits of digestate on FW fermentation, improving pH stability, increasing microbial diversity, maintaining a low oxidation reduction potential (Wang et al., 2021), or simply being a process water source for LA fermentation following pre-treatment (Zhang et al., 2019). While these reports are promising, they seeded LA fermentation with waste activated sludge (Wang et al., 2021) or a specific strain of LA bacteria (Zhang et al., 2019). However, these inoculum sources either provide resilience in terms of a diverse microbial community (i.e., waste activated sludge), or the targeted performance of a pure culture, but not both. Instead, an adapted inoculum would more likely be used in future FW biorefinery concepts that can reliably produce high LA concentrations, as well as having adequate microbial diversity to accommodate imminent process changes. For this reason, it would be vital to understand the impact of digestate for an adapted mixed culture inoculum.

To address the above knowledge gaps, this study assessed the effect of sucrose and N addition (as digestate or NH_4_Cl) on FW LA fermentation, for an acclimatised inoculum, sourced from the first-stage of a commercial two-stage FW AD facility. The study aimed to resolve individual and combined effects of substrate availability (via sucrose addition) and N supplementation, and the distinct effects of N source as digestate or NH_4_Cl. The impact on microbial community and fermentation pathways were also explored. The aim was to improve LA fermentation from FWs to enable future LA-AD biorefinery concepts.

## 2 Methods

### 2.1 Substrate and inoculum

A synthetic mixed FW feedstock was used in this study and prepared following the recipe of Capson-Tojo et al. (2017) (Supplementary Table S1). Preparation of the FW included maceration, blending, and screening as described by Bühlmann et al. (2022). The synthetic substrate was stored overnight at 1–4°C before use. As per Bühlmann et al. (2021), the inoculum was obtained from the acidic fermentation stage (first-stage) of a commercial-scale two-stage AD facility treating FW. Anaerobic digestate was sourced from an anaerobic digester at the same facility. The inoculum and digestate were stored at 1–4°C before use. Compositional analysis of the prepared synthetic FW, inoculum, and digestate was conducted at the Analytical Reference Laboratory (Perth, Australia) using standard methods (Supplementary Table S2). Reagent grade sucrose (Chem-Supply, Australia; SA030) and analytical reagent grade ammonium chloride (Chem-Supply, Australia; AA049) were used as carbohydrate and model N source in the experiments, respectively.

### 2.2 Batch fermentation tests

Batch fermentation tests were performed in glass serum vials (250 mL). Vials were filled with 20 mL inoculum and 180 mL synthetic FW, and digestate (see below) or tap water up to a total working volume of 234 mL. Sucrose crystals were added at dosages of 0, 43, 107, or 150 g_sucrose_·L_mixture_^−1^ to align with similar conditions of relevant past studies (Reddy et al., 2015). In line with Zhang et al. (2020b) (Section 1), fermentation vessels were supplemented with N at 0, 300, or 400 mgN·L_mixture_^−1^ (excluding background), added as NH_4_Cl powder (Chem-Supply; AA049) or digestate. Levels in each test vial was set by a bi-factorial experimental design, assessing the effects of sucrose and N addition. The tests were conducted in four blocks (Supplementary Table S3), which did introduce an additional time factor, due to progressive aging of the inoculum from block 1 to 4, and this was added as a separate variable in the subsequent analysis. Following reagent addition (as relevant), the test vessels were sealed with a screw top cap plugged with a butyl rubber septum and the headspace was purged with high purity nitrogen, and the fermentation mixtures were adjusted to pH 6.0 and maintained at this pH by the method previously reported elsewhere (Bühlmann et al., 2022). The vessels were then incubated at 50°C for 5 days. A previous study by the authors identified this test pH and temperature as being preferred for LA fermentation by the same adapted inoculum (Bühlmann et al., 2022). pH was measured using a calibrated pH meter (Rowe Scientific, Australia; IP1400 and IP1163). Liquid samples were periodically collected for measurements of LA and other volatile fatty acids (VFAs) (Section 2.3). For this, the vessel was inverted, and a 5 mL sample was extracted and stored in 15 mL centrifuge vials for a maximum of 2 days at 1–4°C prior to analysis (Section 2.3). At the end of fermentation (5 days), an additional 10 mL sample was taken and immediately stored at −20°C for DNA sequencing (Section 2.4).

### 2.3 Analytical methods

Total solids (TS) and volatile solids (VS) were measured according to Standard Methods (APHA, 1995). Prior to organic acid analysis, each liquid sample was centrifuged at 10,000 g for 10 min and the pellet discarded while the supernatant from the centrifuged sample was collected for analysis. To ensure the organic acid concentrations were within measurement range, predetermined quantities of deionised water were used to dilute the liquid sample. The diluted mixture was then filtered through a 0.45 mm PES Millipore® filter before measurement by HPLC (Bühlmann et al., 2021). LA selectivity was calculated using Eq. 1 after first converting acid concentrations from g·L^−1^ to gCOD·L^−1^ using theoretical COD to mass ratios.

$$LA\left(\%\right)=\frac{C_{LA}}{C_{LA}+C_{SA}+C_{AA}+C_{PA}+C_{BA}}$$ (1)

where *C*_*LA*_, *C*_*SA*_, *C*_*AA*_, *C*_*PA*_, and *C*_*BA*_ denote the LA, succinic acid, acetic acid, propionic acid, and butyric acid concentration (gCOD·L^−1^), respectively.

### 2.4 DNA extraction and amplification

Prior to DNA extraction, the frozen whole liquid samples collected on fifth day of fermentation were thawed and vortexed for 15 s. Detailed methods describing DNA extraction, amplification, and screening can be found elsewhere (Bühlmann et al., 2022). The extracted DNA was sequenced at the Australian Centre for Ecogenomics (ACE), The University of Queensland (Brisbane, Australia), on the Illumina® Mi-seq platform.

### 2.5 Bioinformatics

#### 2.5.1 Taxonomy analysis

Taxonomic assignment used Mothur v1.46.1 (Schloss et al., 2009) using a slightly modified operating procedure. The Silva database (Release v132) was used to assign operational taxonomic units to the processed sequences based on 97% similarity. Detailed description of the methods undertaken for the taxonomy analysis can be found elsewhere (Bühlmann et al., 2022).

#### 2.5.2 Phylogenic investigation of communities by reconstruction of unobserved states (PICRUSt)

For PICRUSt, sequences were again processed using Mothur 1.46.1 (as above) and were assigned GreenGene (gg_13_5) operational taxonomic units based on 97% similarity. For this study, NSTI values ranged from 0.06 ± 0.003 to 0.12 ± 0.019 with an average of 0.099 ± 0.019 s d which is lower than the threshold (0.15) used to indicate similarity with the reference genome database and similar to those for environmental communities (Langille et al., 2013; Louvado et al., 2020). The KEGG database was used to identify all genes (KEGG, 2022).

### 2.6 Data analysis and statistics

As the inoculum naturally contained LA and other organic acids, all acid yields and concentrations presented below are displayed as net values (i.e. measured values minus the initial concentration at time *t* = 0). All measured data is presented as the mean ± 95% confidence interval (calculated using a two-tailed student t-test) unless otherwise stated. Acid yields were normalized with respect to the initial VS of FW and sucrose added (not including VS from added inoculum or digestate). The rate of LA formation and maximum LA yield were estimated using a first-order plus lag model (Eq. 2).

$$P\left(t\right)=P_{\max}\left(1-\exp\left(-k(t-\theta \right)\right)$$ (2)

where *P* (*t*) is LA yield (g_LA_·g_VS_^−1^) at time *t* (h), *P*_*max*_ is the maximum LA yield (g_LA_·g_VS_^−1^), *k* is the first-order rate constant (h^−1^), and θ is an initial time lag (h). This analysis was conducted in AQUASIM 2D (Reichert, 1994) and included all data up to the visually identified maximum measured LA yield. Parameter uncertainty was estimated at the 95% confidence limit based on a two-tailed *t*-test on parameter standard error around the optimum, as determined by AQUASIM 2D. The coefficient of determination (*R* ^2^) of the model fits were calculated in Microsoft Excel. Response surface methodology (RSM) was used to identify single and interactive effects of N supplementation and sucrose addition on the maximum LA concentration and rate of LA formation (*k* values from Eq. 2). Independent variables were sucrose (*X*_*S*_), N_dosage (*X*_*N*_), and the N_source (*X*_*NS*_). The raw triplicate data of measured LA concentration (individual observations) and the model estimates of *k*, were the response variables in separate analyses. For the statistical analysis, the numerical independent variables were normalised linearly (Supplementary Table S3), to ensure each predictor had an equal weighting. N source was included as a categorical variable (*X*_*NS*_; N_Source) in the model (0 = NH_4_Cl, 1 = Digestate). As the tests were conducted in runs in time sequence (4 blocks in total), a block factor (*R*_*B*_) was included within the regression analysis as a continuous factor (1–4) to test for aging of the inoculum (Table 1). The standard scores were fitted to a second order regression model (Eq. 3) via least squares regression analysis, as follows:

$$Y=\beta_{0}+R_{B}X_{B}+\left(\beta_{S}X_{S}\right)+\left(\beta_{N}X_{N}\right)+\left(\beta_{NS}X_{NS}\right)+\left(\beta_{S\_N}X_{S}X_{N}\right)+\left(\beta_{S\_NS}X_{S}X_{NS}\right)+\left(\beta_{N\_NS}X_{N}X_{NS}\right)+\left(\beta_{S^{2}}X_{S}^{2}\right)+\left(\beta_{N^{2}}X_{N}^{2}\right)$$ (3)

where *β*_*0*_ is an intercept, *β*_*S*_, *β*_*N*_, and *β*_*NS*_ are linear terms, *β*_*S_N*_, *β*_*S_NS*_, and *β*_*N_NS*_ are two-way interaction terms, and *β*_*S^2*_ and *β*_*N^2*_ are squared effects. Model parameters were determined using the RSM function in R (R Development Core Team, 2022). To avoid overfitting and ensure the most significant parameters remained within the model, the step() function was applied to sequentially remove parameters from the model as previously described (Bühlmann et al., 2021). The 95% confidence intervals for each parameter estimate were determined using confint() in R, and 95% confidence intervals for the model predictions were determined using the predict() function in R. To assess the effects of N supplementation and sucrose addition on other measured organic acids, microbial community composition, and putative metabolic pathways, the RSM described above was further applied to individual VFA concentrations achieved at the visually selected maximum LA concentration, the relative abundance of genera (>1%), and select genes related to LA formation, as respective response variables in separate analyses. The relative abundance of all genes included in the analysis was arbitrarily multiplied by a factor of 1,000 to improve the sensitivity of the model fit. Predictor variables remained unchanged from that described above. To further explore the effects of sucrose, ammonium, and digestate on the product spectrum, a principal component analysis (PCA) was conducted on the VFA concentrations at the peak LA concentration using the prcomp() function in R with scale = T.

**TABLE 1:** Kinetic parameters for the first-order model. Errors (±) represent 95% confidence intervals.

Block	Sucrose (g·L^−1^)	N (mgN·L^−1^)^a^	Max. Time (h)^b^	Max. LA (g_LA_·L^−1^)	Net. Yield (g_LA_·g_VS_^−1^)^c^	k (h^−1^)	Lag phase (h)
1	0	0	60	25.7 (±2.2)	0.63 (±0.06)	0.08 (±0.03)	9.4 (±2.0)
1	107	0	120	61.7 (±3.2)	0.45 (±0.01)	0.04 (±0.01)	4.3 (±0.8)
1	43	300	72	51.7 (±4.6)	0.62 (±0.03)	0.08 (±0.02)	7.4 (±1.4)
1	150	300	120	60.9 (±5.7)	0.35 (±0.01)	0.04 (±0.01)	6.4 (±1.4)
1	0	400	48	26.1 (±0.6)	0.64 (±0.02)	0.13 (±0.02)	4.1 (±0.7)
1	107	400	120	66.5 (±9.7)	0.50 (±0.01)	0.03 (±0.01)	7.6 (±1.0)
2	43	0	72	44.7 (±3.7)	0.60 (±0.08)	0.04 (±0.02)	10.6 (±2.2)
2	150	0	120	56.2 (±6.7)	0.33 (±0.01)	0.03 (±0.01)	8.9 (±0.9)
2	0	300	84	29.7 (±0.6)	0.69 (±0.03)	0.08 (±0.02)	6.3 (±2.1)
2	107	300	120	66.9 (±5.2)	0.49 (±0.01)	0.04 (±0.01)	7.2 (±0.9)
2	43	400	72	52.8 (±4.9)	0.66 (±0.03)	0.05 (±0.01)	8.5 (±1.3)
2	150	400	96	59.9 (±9.7)	0.35 (±0.01)	0.04 (±0.01)	7.7 (±1.0)
3	0	300_D_	48	28.5 (±2.4)	0.69 (±0.05)	0.11 (±0.04)	9.0 (±1.2)
3	43	300_D_	60	51.3 (±7.0)	0.62 (±0.03)	0.09 (±0.02)	8.1 (±1.2)
3	107	300_D_	72	63.7 (±15.9)	0.49 (±0.03)	0.05 (±0.01)	7.3 (±2.1)
3	0	400_D_	60	29.9 (±0.5)	0.72 (±0.05)	0.13 (±0.05)	5.3 (±1.2)
3	43	400_D_	60	52.1 (±8.6)	0.64 (±0.02)	0.09 (±0.02)	8.9 (±1.0)
3	150	400_D_	84	68.2 (±8.1)	0.40 (±0.02)	0.05 (±0.01)	7.4 (±1.4)
4	150	300_D_	96	47.2 (±2.9)	0.29 (±0.01)	0.04 (±0.01)	17.1 (±1.1)
4	107	400_D_	72	51.3 (±15.6)	0.38 (±0.02)	0.07 (±0.02)	15.2 (±1.5)

^a^ N source is indicated as either NH_4_Cl (no subscript) or digestate (subscript D).

^b^ Corresponds to the time at which the LA concentration was at its maximum value.

^c^ Yield on FW and sucrose.

## 3 Results and discussion

### 3.1 Effect of sucrose and nitrogen addition on lactic acid production

**FIGURE 1:**
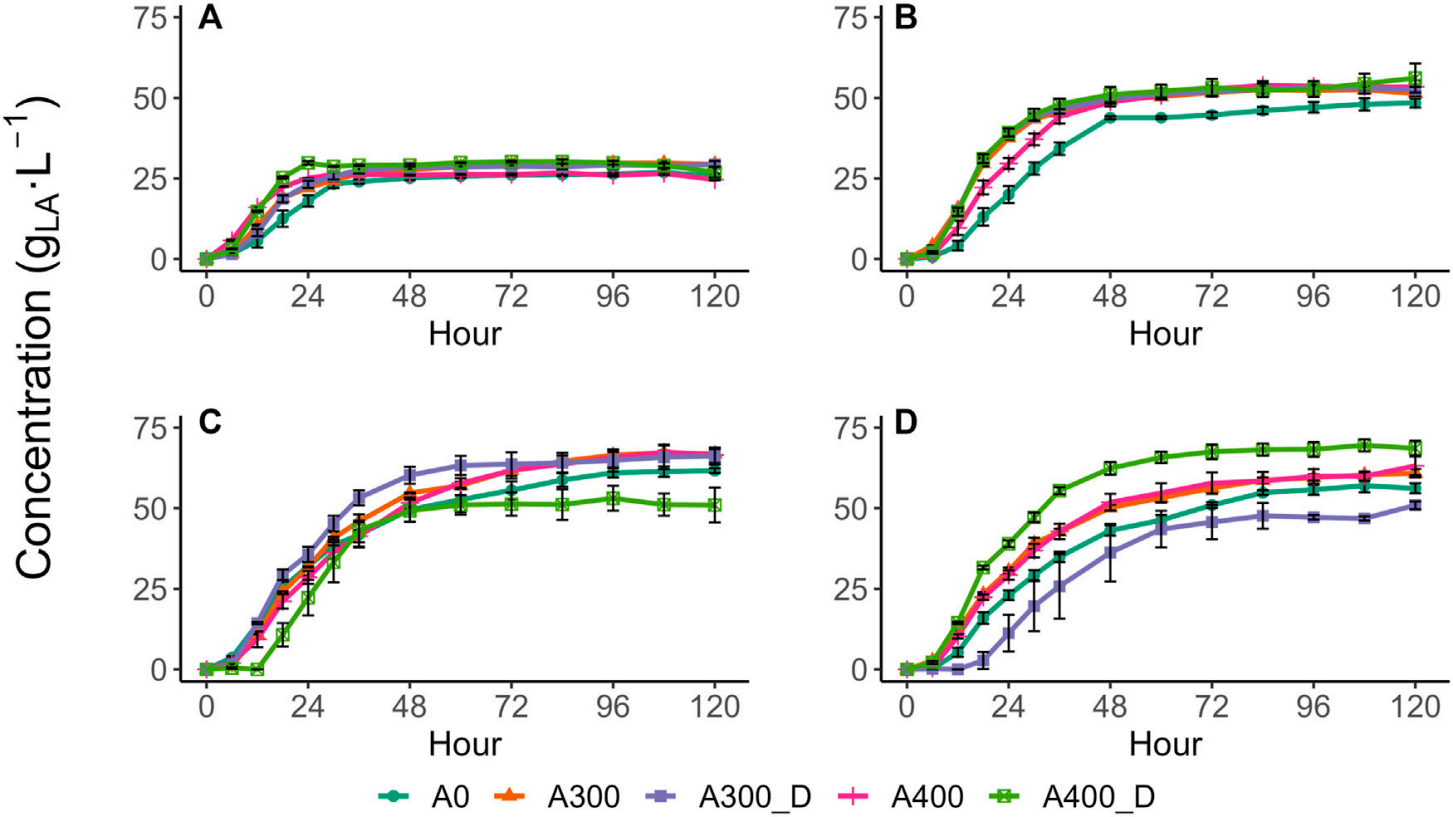
Lactic acid production profiles with sucrose amendments of **(A)** 0, **(B)** 43, **(C)** 107, and **(D)** 150 g⋅L^−1^. Values are presented as the mean of triplicates ± the standard error.

All test conditions showed similar LA production profiles, with the LA concentration initially rising rapidly to an asymptotic final value, with minimal to no subsequent LA depletion observed over the 120 h test period (Figure 1) confirming the test conditions outlined by Bühlmann et al. (2022) promoted LA accumulation. Consequently, all tests were appropriately described by 1st order kinetics with an initial time lag (Table 1). With no sucrose or N addition, LA accumulated rapidly within the first 24 h, and then slowed significantly, reaching a maximum yield of 0.63 g_LA_·g_VS_^−1^ by 60 h (Table 1). This yield was similar to those reported by studies conducted at similar conditions e.g., 0.57 g_LA_·g_VS_^−1^ (Yang et al., 2022), 0.58 g_LA_·g_VS_^−1^ (Akao et al., 2007), and 0.55 g_LA_·g_VS_^−1^ (Bühlmann et al., 2022).

The final simplified RSM models (Supplementary Figures S1, S2) described the observed data well, having an adjusted *R* ^2^ of 0.89 and 0.88 (Table 2). No two-way interactions were retained by the step() function, except for a single interaction term within the rate model, albeit that its coefficient estimate was not found to be significant (i.e., not significantly different from zero) (Table 2).

**TABLE 2:** Simplified RSM model parameters with associated 95% confidence intervals.

Variable	Symbol	LA concentration model	k (Rate) model
Intercept	*β*_*0*_	32.32 (±4.88)***	0.09 (±0.02)***
Block	*R*_*B*_	−4.97 (±2.69)***	−0.01 (±0.01)
Sucrose	*β*_*S*_	95.52 (±12.47)***	−0.12 (±0.06)***
N_Amount	*β*_*N*_	5.21 (±3.62)**	0.03 (±0.02)*
N_Source	*β*_*NS*_	5.99 (±5.43)*	0.03 (±0.02)*
Sucrose^2^	*β*_*S^2*_	−63.91 (±12.02)***	0.08 (±0.05)**
N_Amount^2^	*β*_*N^2*_	—^a^	—^a^
TWI(Sucrose:Ammonia)	*β*_*S_N*_	—^a^	−0.03 (±0.04)
TWI(Sucrose:N_Source)	*β*_*S_NS*_	—^a^	—^a^
TWI(Ammonia:N_Source)	*β*_*N_NS*_	—^a^	—^a^
Adj.R^2^	—	0.89	0.88

^a^ Removed by step () function (Section 2.6.1).

***=(*p* < 0.001), **=(*p* < 0.01), *=(*p* < 0.05).

Sucrose displayed a strong positive linear effect and a strong negative second-order effect on LA concentration (Table 2), indicating that LA concentration was increased by sucrose addition up to a certain dosage (Table 1), but higher dosages led to a reduction in LA concentration, likely due to substrate inhibition. In contrast, sucrose displayed a strong negative linear effect on LA formation rate (*k*), indicating rate inhibition at all levels. Sucrose also had a minor positive second-order effect on *k* (Table 2). *N_Amount* retained a positive linear effect on both LA concentration (and yield) and *k*, with the model estimating an incremental concentration increase of 5.2 ± 4.6 g·L^−1^ LA at the highest level (400 mgN·L^−1^) compared to a modelled base case with no sucrose or N addition (i.e., 27.3 ± 3.4 gLA·L^−1^). Similar studies by Zhang et al. (2020b); Zhang et al. (2020a) outlined a 2–2.4 fold increase in LA concentration resulting from N supplementation using NH_4_Cl, much higher than that observed in the current study (max 1.2-fold). This difference may be, at least partially, due to different inoculum sources and FW utilised. The seed material has been suggested to be a crucial in the development of relevant metabolic pathways, production of LA, and evolution of competing biological processes (Wang et al., 2014; Tang et al., 2017; Arras et al., 2019). Both Zhang et al. (2020b) and Zhang et al. (2020a) utilised waste activated sludge for inoculation and reported NH_4_Cl significantly increasing the relative abundance of LA producers. However, the adapted mixed inoculum utilised in the current study had LA bacteria naturally dominant (Section 3.3), even in the 0 N test (Section 3.3). The use of an adapted inoculum could have promoted LA production, leading to a lower overall response from N addition as NH_4_Cl. A lower background N concentration (more limited N conditions) could also have caused the larger response to N observed by Zhang et al. (2020b); Zhang et al. (2020a) but they did not report compositional data for their FW, so background N levels in their study could not be estimated. The RSM model showed that digestate led to a 1.3 ± 4.5 g L^−1^ change in the LA concentration, compared to the modelled base case, and at the highest digestate level resulted in an increased *k* of 0.13 ± 0.01 h^−1^, compared to the base case of 0.08 ± 0.02 h^−1^. Similar to the current study; Wang et al. (2021) outlined that industrial digestate improved LA fermentation when utilised at a ratio of 0.2 L_digestate_·L_feedstock_^−1^ (current study used a ratio of 0.19 L_digestate_·L_feedstock_^−1^, at 400 mgN·L^−1^). While digestate contains high concentrations of NH_4_^+^-N, its complex matrix also contains various other nutrients (Supplementary Table S2) and additional fermentative bacteria which may further aide LA fermentation or increase substrate utilisation for alternative organic acids. The second-order effect for N was not significant in either of the RSM models (Table 2), suggesting that, unlike for sucrose, inhibitory concentrations for NH_4_Cl and digestate were not reached in the current study. Previous research by Zhang et al. (2020b) outlined a reduction in LA production with NH_4_^+^-N supplementation above 500 mgN·L^−1^, which is higher than the maximum added dose in the current study (400 mgN·L^−1^). Limited research is available exploring LA fermentation with added digestate, however, it has been suggested that excessive ammonia-N, zinc, iron, sulphur, and manganese within digestate could inhibit *Lactobacillus casei* during batch LA fermentation from starch, when the digestate is used as a process water source (Zhang et al., 2019). Comparably, Wang et al. (2021) suggested that excessively high dosages of digestate would alter fermentation pathways, lowering LA selectively; however, these same authors did not report any inhibition of fermentation, possibly because of relatively lower digestate dosages and a mixed culture utilised for fermentation in their study.

Overall, the net LA yield was highest at the lowest sucrose level but was generally improved, albeit by small increments, by N supplementation at all N dosage rates (Table 1). The N effect is supported by RSM results in Table 2. With NH_4_Cl, at the lowest sucrose level, the net LA yield was at its maximum for the FW sucrose mixture, regardless of N dosage. With digestate, similar net yields were achieved at the lowest sucrose level, albeit higher variability in the measured max LA concentration were noted (Table 1). At the higher sucrose dosage of 107 g L^−1^, the net LA yield reduced, possibly due to the previously mentioned substrate inhibition, however, all yields were similar regardless of N dosage or source, apart from 400 mgN·L^−1^ with digestate, which saw a 24% reduction in net LA yield, as compared to 400 mgN·L^−1^ with NH_4_Cl. At the highest sucrose level, net yields were similar across treatments (Table 1), apart from 300 mgN·L^−1^ with digestate, which saw a 17% reduction in net yield, as compared to 300 mgN·L^−1^ as NH_4_Cl.

### 3.2 Product spectrum

Due to the complex composition of FW and presence of a mixed microbial community (Section 3.3), fermentation of mixed FW for LA will also lead to the production of competitor organic acids through the competitive uptake of available substrate or by the consumption of LA as the substrate, resulting in a lower LA yield/selectivity (Arras et al., 2019). To minimise downstream processing costs and increase LA output, it is important to tune LA fermentation to minimise the production of competitor acids wherever possible.

**FIGURE 2:**
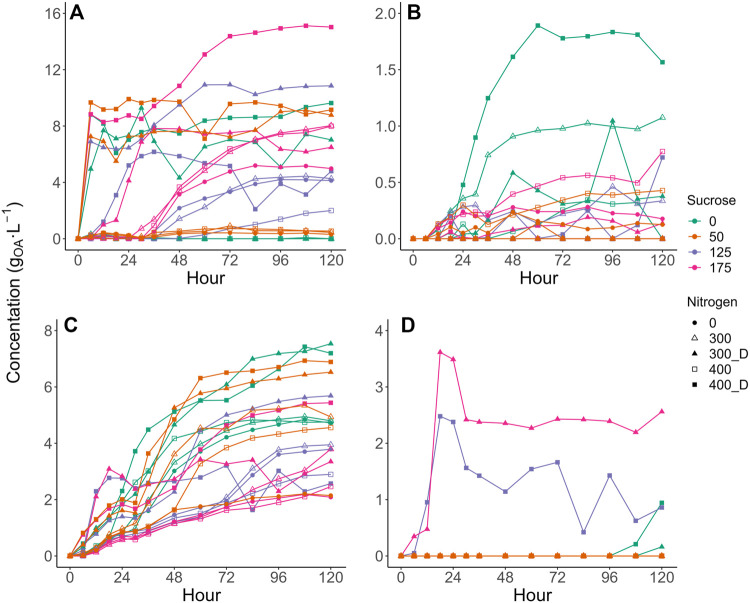
Production curves of **(A)** Acetic acid, **(B)** Succinic Acid, **(C)** Propionic Acid, and **(D)** Butyric Acid. Values are presented as the mean of triplicates. Error bars were removed to improve readability. Please see Supplementary Figure S9 in the Supplementary Material for expanded plot with error bars.

The observed production of various competitor VFAs varied dynamically with sucrose, NH_4_Cl, and digestate addition. Acetic acid and propionic acid production profiles differed the most between treatments, displaying variations in both apparent production rate and maximum concentration achieved (Figures 2A, C). In contrast, succinic and butyric acid production generally followed similar production profiles with different treatments with production peaking in select treatments (Figures 2B, D).

**FIGURE 3:**
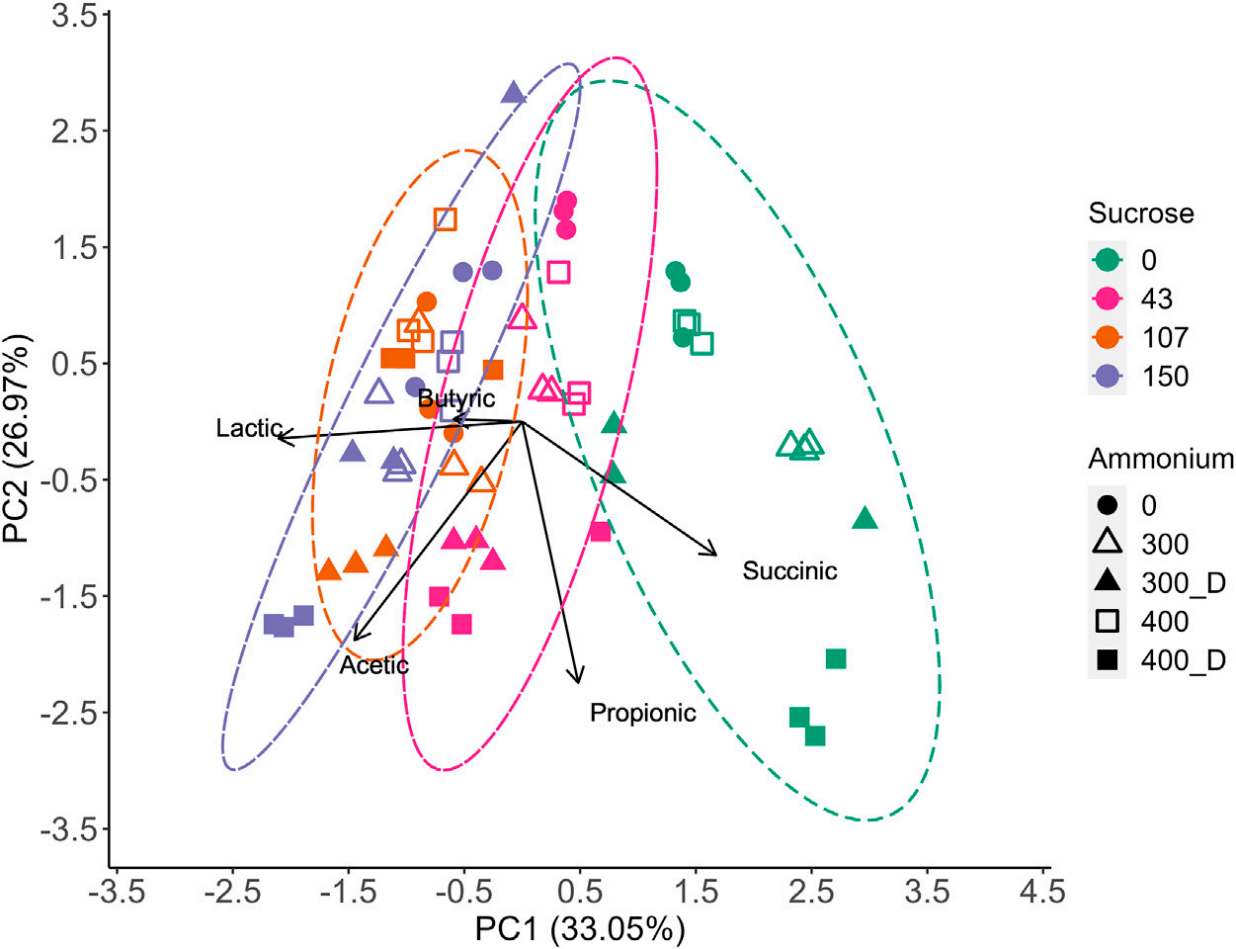
Principal Component Analysis (PCA) showing the influence of sucrose, NH_4_Cl, and digestate on the product spectrum at the peak LA concentration for each treatment level. Ellipses represent the 95% confidence interval for the sucrose impact on the product spectrum.

As LA would likely be recovered at an LA-AD facility at its peak concentration (Table 1), it was appropriate to carry out an analysis of variable effects on VFA concentrations measured for samples of each experiment for which LA concentration was at its maximum. The resulting RSM model (Table 3) interestingly showed that while *N_Source* was retained within all models by the step() function, its effect was not significant in the acetic and propionic acid models. However, acetic acid was generally observed to be higher in the digestate treatments (Figure 2A; Supplementary Table S4), indicating digestate played some role in promoting acetic acid production. Therefore, a PCA was conducted on the same data set (Figure 3) to further explore the variable impacts on the product spectrum.

**TABLE 3:** Simplified RSM model parameters for competitor VFAs given with ±95% confidence intervals.

Variable	Succinic	Acetic	Propionic	Butyric
Intercept	−0.19 (±0.37)	3.18 (±2.47)*	4.49 (±1.1)***	−0.74 (±0.61)*
Block	0.37 (±0.19)***	−2.10 (±1.40)**	−1.13 (±0.52)***	0.52 (±0.36)**
Sucrose	−1.87 (±0.88)***	—^a^	1.39 (±2.66)	−0.08 (±0.54)
N_Amount	0.20 (±0.26)	0.40 (±1.95)	7.05 (±3.54)***	—^a^
N_Source	−1.03 (±1.03)*	3.90 (±7.7)	−2.69 (±3.82)	−0.95 (±0.76)*
Sucrose^2^	1.69 (±0.83)***	6.21 (±1.67)***	−1.60 (±2.30)	—^a^
N_Amount^2^	—^a^	—^a^	−6.02 (±3.57)**	—^a^
TWI(Sucrose:Ammonia)	—^a^	—^a^	−1.37 (±1.66)	—^a^
TWI(Sucrose:N_Source)	−1.04 (±0.46)***	—^a^	—^a^	1.08 (±0.86)*
TWI(Ammonia:N_Source)	1.12 (±1.1)*	5.68 (±8.33)	6.47 (±4.2)**	—^a^
Adj.*R* ^2^	0.54	0.67	0.6	0.34

^a^ Removed by step () function (Section 2.6.1).

***=(*p* < 0.001), **=(*p* < 0.01), *=(*p* < 0.05).

The PCA displayed a strong relationship between sucrose addition and acetic acid production, which is in agreement with the RSM model (Table 3). However, while *N_Source* was non-significant within the model, a clear relationship between NH_4_Cl and digestate was observed, with digestate increasing acetic acid production.

The highest competitor VFA levels (22.1 gCOD·L^−1^) were observed at 150 g⋅L^−1^ sucrose and 400 mgN·L^−1^ as digestate. In contrast only 3.9 gCOD_VFA_·L^−1^ of VFAs were produced with 43 g L^−1^ sucrose and no N added, increasing to only 5.5 gCOD·L^−1^ with 400 mgN·L^−1^ as NH_4_Cl. Compared to the modelled base case (i.e., no sucrose, no added N), LA selectivity improved at these conditions, achieving 91–92% LA as compared to 83% at base case. All digestate treatments produced more competitor VFAs than those without added N or with NH_4_Cl (Supplementary Table S4). However, like NH_4_Cl, higher sucrose dosages increased the production of LA (Figure 3) and, while competitor VFA production also increased, sucrose generally increased LA production to a larger extent than that of competitor acids (Tables 2, 3), increasing the overall LA selectivity. Previous work by Zhang et al. (2020b) suggested proteins present within waste activated sludge can be a substrate for VFA production, elevating undesired metabolite levels, which may have occurred in this study. However, as the digestate was not sterilised in the current study, alternative fermentative bacteria introduced with the digestate likely competed with LA bacteria for substrate and contributed to the observed increase in VFA production (Section 3.3). Butyric acid production generally remained low for all treatments, except for some digestate containing vessels with sucrose at 107–150 g·L ^−1^ (Supplementary Figure S7). At 400 mgN·L^−1^ without sucrose, butyric acid production appeared to be accompanied by a slight reduction in the LA yield at the end of fermentation (Supplementary Figure S3E), suggesting the initiation of LA consumption for butyric acid, which as been previously observed for this inoculum (Bühlmann et al., 2022). Previous research has suggested that the formation of butyric acid from LA may be related to substrate availability (Detman et al., 2019; Hoelzle et al., 2021), with the addition of substrate shown to prevent the conversion of LA to butyric acid (Hoelzle et al., 2021). While it cannot be confirmed that butyrate production was prevented through sucrose addition in the current study, butyric acid was not detected with sucrose and NH_4_Cl addition and was only observed in some digestate containing treatments (Figure 2D), which could be related to changes in microbial community composition (Section 3.3).

### 3.3 Microbial community analysis

The most abundant phyla across all treatments were Firmicutes (66–99%), with other minor phyla including Actinobacteria (0.2–29%), Bacteroidetes (0.0–2.4%), Euryarchaeota (0–2.0%), Chloroflexi (0.0–1.4%), and Thermotogae (0–0.8%) (Supplementary Figure S7). While all phyla were detected in nearly all treatments, digestate likely acted as a secondary inoculum. For example, Bacteroidetes, Thermotogae, Actinobacteria, Euryarchaeota, and Chloroflexi were primarily enriched in the digestate treatments (Supplementary Figure S7). Chloroflexi, and Bacteroidetes are commonly found within FW AD systems (St-Pierre and Wright, 2014; Buhlmann et al., 2018), and were likely inoculated when digestate was added to test vessels. Thermotogae have been reported to form a syntrophic relationship with hydrogenotrophic methanogens for the oxidation of acetate during methanogenesis at high total ammoniacal N concentrations (Li et al., 2016). The digesters from which digestate was sourced in the current study, have been reported to operate at elevated total ammoniacal N concentrations (Buhlmann et al., 2018), which could have caused an increased relative abundance of Thermotogae in the digestate treatments.

**FIGURE 4:**
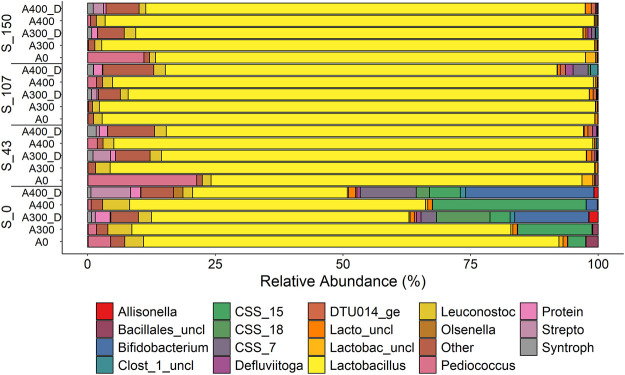
Relative abundance of microbial genera (>1%) following 5-days of FW fermentation supplemented with sucrose and N (NH_4_Cl or digestate). Sucrose level is denoted by an “S” followed by the initial supplement concentration, while supplemented N is denoted by an “A” and N added as digestate is denoted by “D”. The names of various genera have been shorted to fit them within the legend. Full names are as follows: Bifid (Bifidobacteriaceae), Clost_1 (Clostridiaceae_1), CSS (Clostridium sensu stricto), Lacto (Lactobacillales), Lactobac (Lactobacillaceae), Protein (Proteiniphilum), Strepto (Streptococcus), and Syntroph (Syntrophaceticus). All genera containing “uncl” were unclassified.

*Lactobacillus* was the dominant genus within all treatments but showed a reduced relative abundance in the RSM model when digestate was added without sucrose (Supplementary Table S5), down to 50% and 30% with digestate dosages of 300 and 400 mgN·L^−1^, respectively (Figure 4). *Clostridium Sensu Stricto 15* (*CSS_15*) proliferated with the addition of 400 mgN·L^−1^ as NH_4_Cl, while *Bifidobacterium*, *CSS_18*, and *Proteiniphilum*, primarily grew in digestate containing environments without sucrose (Sucrose and N_Source effects, Supplementary Table S5). Research detailing the metabolic process of *CSS_15* are limited, however, *Clostridium* include a variety of bacteria which are specialised in utilising multiple sugars to generate methanogenic precursors, such as acetate, butyrate, carbon dioxide, and hydrogen (Song et al., 2021). *Bifidobacterium* form short chain fatty acids (e.g., LA and acetate) from carbohydrates and may form a syntrophy with *Clostridium* for butyrate formation (Xiong et al., 2019), which may have occurred in this study (Section 3.2). *Proteiniphilum* plays an important role in protein degradation and has been isolated from biogas plants, particularly those treating FW, brewery waste, and wheat straw (Orellana et al., 2022), such as the plant from where the digestate was sourced (Bühlmann et al., 2021).

For both NH_4_Cl and digestate treatments, sugar addition at any level significantly suppressed the growth of the flanking community and promoted the growth of *Lactobacillus* (Supplementary Table S5; Figure 4). While research exploring sugar supplementation during FW fermentation could not be found, previous studies have reported that higher substrate concentrations promote LA production and the growth of LA bacteria during FW fermentation (Pu et al., 2019; Simonetti et al., 2021).

### 3.4 Functional gene analysis

To better understand the impact of sucrose and N supplementation on metabolic pathways for LA production, a conceptual pathway diagram was constructed based on various relevant metabolic pathways (Supplementary Figure S8). The resulting predicted genes from the PICRUSt analysis were then utilised to explore the effect of sucrose and N addition on relevant microbial degradation pathways. The *in-silco* prediction suggested LA production likely occurred through multiple pathways working in tandem. The abundance of Lactate dehydrogenase (*LDH*) tended to increase with the addition of digestate (*p* < 0.001; Supplementary Table S6) which aligns with the increased LA concentration associated with those treatments (Section 3.1). Genes utilised within the glycine, serine and threonine metabolism associated with the production of methylglyoxal, namely *AOC3* and *MAO*, fluctuated with the addition of sucrose and N (Supplementary Table S6). *AOC3* was primarily enriched with NH_4_Cl addition, though the combined addition of digestate and sucrose increased the relative abundance of this gene (*p* < 0.001). In contrast, the combination of sucrose and digestate tended to reduce the relative abundance of *MAO* (*p* = 0.04; Supplementary Table S6). Methylglyoxal is a toxic compound produced from glycolysis when glucose consumption surpasses the rate of phosphate uptake during the conversion of dihydroxyacetone-P (Hoelzle et al., 2021). *Lactobacillus* strains generally carry out methylglyoxal detoxification through the production of either acetol (Hydroxyacetone) or 1,2-propanediol (Gandhi et al., 2018), of which a gene related to acetol formation (*yqhD*) was predicted to be present, but tended to reduce in relative abundance with sucrose (*p* < 0.001) or N addition (*p* = 0.03), and this effect was exacerbated with digestate (*p* < 0.001). Detoxification can also occur through the glyoxalase pathway which consists of two enzymes, *GLO1* and *gloB*, which convert the toxic methylglyoxal to D-LA (Jain et al., 2018). The high relative abundance of *gloB* in this study, as compared to alternate LA producing genes (accounting for 81–95% of all identified LA producing genes, i.e., *gloB*, *ldhA*, *dld*, *pct*, *ldh*, and *aldA*), suggests methylglyoxal detoxification could have been a major pathway for LA production at the test conditions. However, the relative abundance of *GLO1* primarily reduced with higher sucrose dosages (*p* < 0.001; Supplementary Table S6). Reduced abundance of this gene may have reduced the capacity of the fermentation system to reduce methylglyoxal, possibly leading to its accumulation to toxic levels at higher sucrose dosages. Such accumulation may have contributed to the reduced LA yields observed at higher sucrose dosages (Section 3.1).

### 3.5 The integrated LA-AD biorefinery

Overall, digestate effectively improved the production of LA from FWs but introduced process variability (increased error bar size; Supplementary Figures S3–S6), increased microbial diversity, and increased the production of alternate organic acids. However, in combination with sucrose, digestate was an effective nutrient source which improved the rate and production of LA (Table 2), while having minimal impact on the microbial community (Figure 4). Furthermore, in an industrial context, digestate could form a valuable process water source to reduce the demand on valuable fresh-water resources, reducing operational costs and environmental impacts from FW processing. Moreover, digestate is commonly considered a liability to many AD facilities, generally requiring pre-treatment (primarily solid-liquid separation) before agricultural land application and tends to be expensive to transport and apply to land because of its moisture content (Turnley et al., 2016; Lu and Xu, 2021). However, the results from the current study have shown that, in combination with sucrose supplementation, digestate recirculation can boost LA fermentation from LA-AD biorefineries for negligible cost (i.e., installation of piping).

In contrast, while sucrose increased the LA concentration and steered fermentation towards LA in the presence of digestate, it is important to explore additional costs associated with its use. Utilising the RSM developed in Section 3.1, the cost to implement sucrose supplementation was estimated at 0.54, 0.85, and 1.33 AUD·kg_LA_^−1^ (based on additional LA produced) for scaled sucrose levels of 0.29, 0.71, and 1, respectively, and assuming a sucrose price of 0.28 AUD·kg^−1^ (0.21 USD·kg^−1^ (Efe et al., 2013)). With the price of LA previously estimated at 2.18 AUD·kg_60wt%_ _LA_^−1^ (1.36 Euro·kg_60wt%_ _LA_^−1^ (Demichelis et al., 2018)) and assuming a recovery efficiency of 51% (Demichelis et al., 2018), the additional cost of sucrose would be easily justified by the value of additional LA product, at all sucrose dosages applied in the current study. Adaptation of the fermentation inoculum to higher sucrose dosages may also improve LA yield on sucrose, thereby increasing the associated economic benefits. However, it is important to note that while fermentation efficiency may have been improved, a fraction of the added sucrose will remain in the fermentation broth. Downstream AD of solid and liquid extraction residues would likely utilise this residual sucrose for methane generation, which can further offset energy requirements of LA separation and recovery, reducing the demand for grid-based fossil-fuel power. This can be important because recovery of LA is known to be energy intensive (Din et al., 2021).

## 4 Conclusion

Overall, the complex FW mixture was effectively fermented for LA production, which was aided by addition of digestate as a relatively low-value N source and supplementing with sucrose as a readily bioavailable substrate. Digestate addition improved both the rate and yield of LA production. However, digestate also increased the microbial diversity which promoted the production of competitor organic acids. Sucrose effectively improved the LA concentration, steered the product spectrum towards LA, and selectively promoted the growth of the desired *Lactobacillus,* while suppressing the flanking community when either NH_4_Cl or digestate were added. A simple evaluation indicated that the value of additional LA produced with sucrose addition outweighed the costs of the sucrose. Overall, the results indicated that an integrated LA-AD biorefinery can effectively implement digestate recirculation without prior pre-treatment or sterilisation, and benefit from sucrose supplementation as a relatively low-value carbon source. This could increase the viability of future LA-AD biorefinery concepts. Future studies should explore the detailed economic impacts of sucrose supplementation and digestate recirculation LA-Ad biorefineries.

## Data Availability

The original contributions presented in the study are publicly available. This data can be found here: [https://www.ncbi.nlm.nih.gov/bioproject/945514].
