# Supplementary material for: Lactic acid production from food waste at an anaerobic digestion biorefinery: effect of digestate recirculation and sucrose supplementation
Source: Front Bioeng Biotechnol. 2023 May 12;11:1177739. doi: 10.3389/fbioe.2023.1177739 (PMC10214416; doi:10.3389/fbioe.2023.1177739)
Supplement: Supplementary file 1 [file DataSheet1.docx]

Lactic acid production from food waste at an anaerobic digestion biorefinery: Effect of digestate recirculation and sucrose supplementation

**Authors:** Christopher H. Bühlmann ^⁎^, Bede S. Mickan, Stephan Tait, Damien J. Batstone, Parisa A. Bahri

* Correspondence:

Christopher Heinz Bühlmann

[Christopherheinz.buhlmann@murdoch.edu.au](mailto:Christopherheinz.buhlmann@murdoch.edu.au)

# **Supplementary material**

**Methods:**

### *S1. Taxonomic analysis*

Taxonomic assignment was carried out by the Australian Centre for Ecogenomics. Primer sequences were removed using cutadapt (version 2.10) with reads which don’t contain primers being discarded (--discard-untrimmed). Poor quality reads were identified and then removed using trimmomatic (version 0.39) sing a sliding window of 4 bases with an average quality score of 15 (SLIDINGWINDOW:4:15). Reads were then cropped at 250 bp (CROP:250) with sequences less than 250 bp being discarded.
Quality controlled forward reads were then processed using QIIME2 (version 2020.11.1) for feature selection, abundance calculations, and taxonomy assignment. Reads were deionised (filtered, dereplicated, and chimeras identified and removed) using DADA2 (--p-trunc-len = 0) with the relative frequencies of each resulting feature being calculated. Taxonomic assignment for each feature was assigned by BLASTing its sequence against the Silva database (release 138, clustered at 99% identity) using the classify-consensus-blast function with default parameters.

### *S2. PICRUSt analysis*

Sequenced data for PICRUSt was processed using Mothur (version 1.46.1) using a slightly modified standard operating procedure (Schloss et al., 2009). Sequences were removed if they did not meet the following quality control: barcode miss match = 1, primer mismatch = 2, ambiguous base calls = 0, minimum quality score Q > 6 or > 25 depending on if forward and reverse reads contained any missing bases, maximum homopolymers length = 8, and maximum length (311) of base pairs per amplicon. Following quality control, retained sequences were pre-clustered to remove any PCR-based bias and singletons. The functions chimera.vsearch() and remove.seqs() in Mothur were used to identify and remove chimeric sequences. Sequences were separately aligned with the Greengenes database (gg_13_5) and assigned to operational taxonomic units (OTU- based taxonomic analysis) based on 97 % similarity.
To determine the dominance of various functional genes, a PICRUSt analysis was performed using the Huttenhower Lab Galaxy server (Harvard, Massachusetts) with the Greengenes OTU identification codes (Langille et al., 2013). The weighted Nearest Sequenced Taxon Index (NSTI) was calculated for each sample to assess the accuracy of the PICRUSt predictions. The weighted NSTI describes the average branch length which separates each OTU in the sample from the referenced genome weighted by the relative abundance of the genome in the sample. NSTI values for this study ranged from 0.06 ± 0.003 to 0.12 ± 0.019 with an average of 0.099 ± 0.019 s.d. The average weighted NSTI for this study is similar to those for environmental communities and lower than the 0.15 threshold used to indicate similarity with the reference genome database (Langille et al., 2013; Louvado et al., 2020). All genes were identified via the KEGG database (KEGG, 2022).

**Figures:**

**
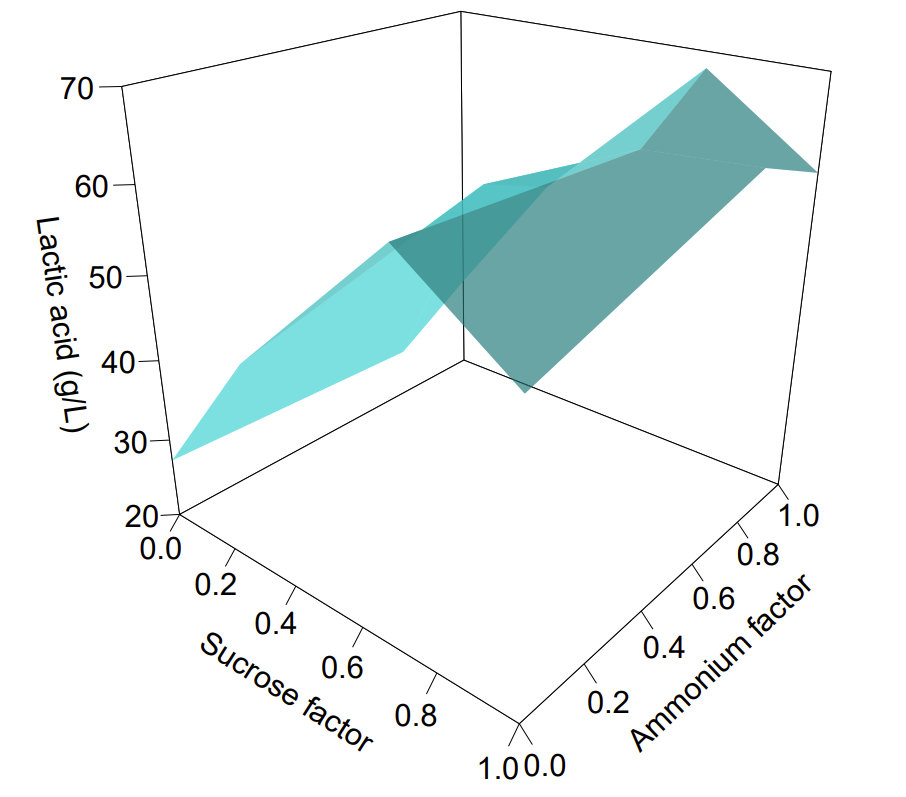
**

**Figure S1:** Optimised RSM plot slowing the impacts of sucrose and NH_4_Cl on lactic acid production from food waste.

**
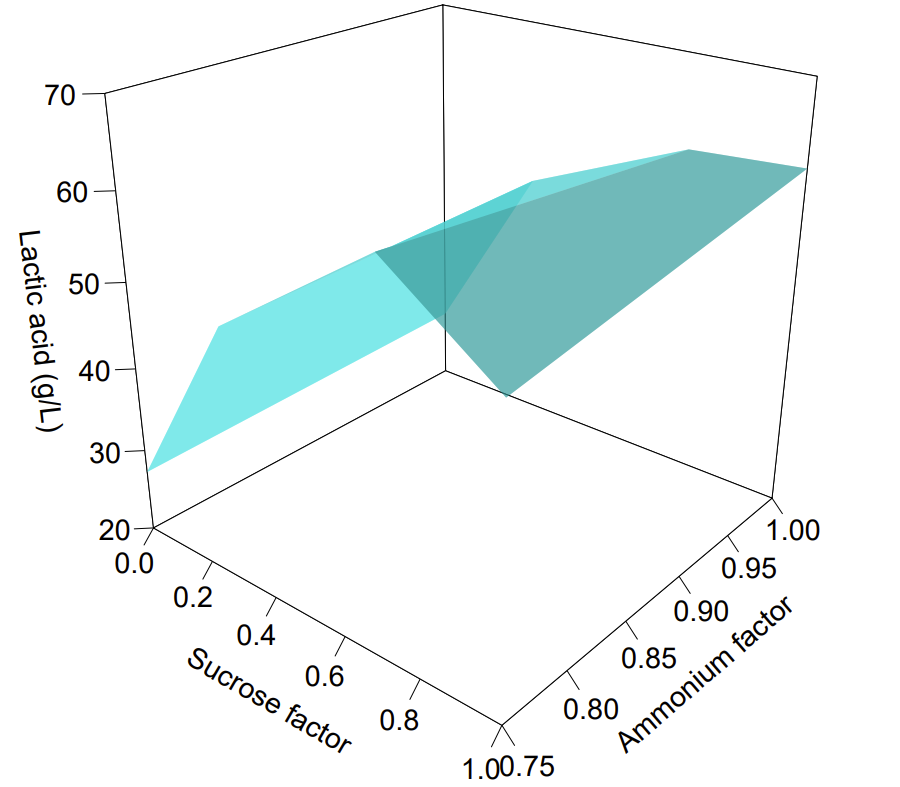
**

**Figure S2:** Optimised RSM plot slowing the impacts of sucrose and digestate on lactic acid production from food waste.


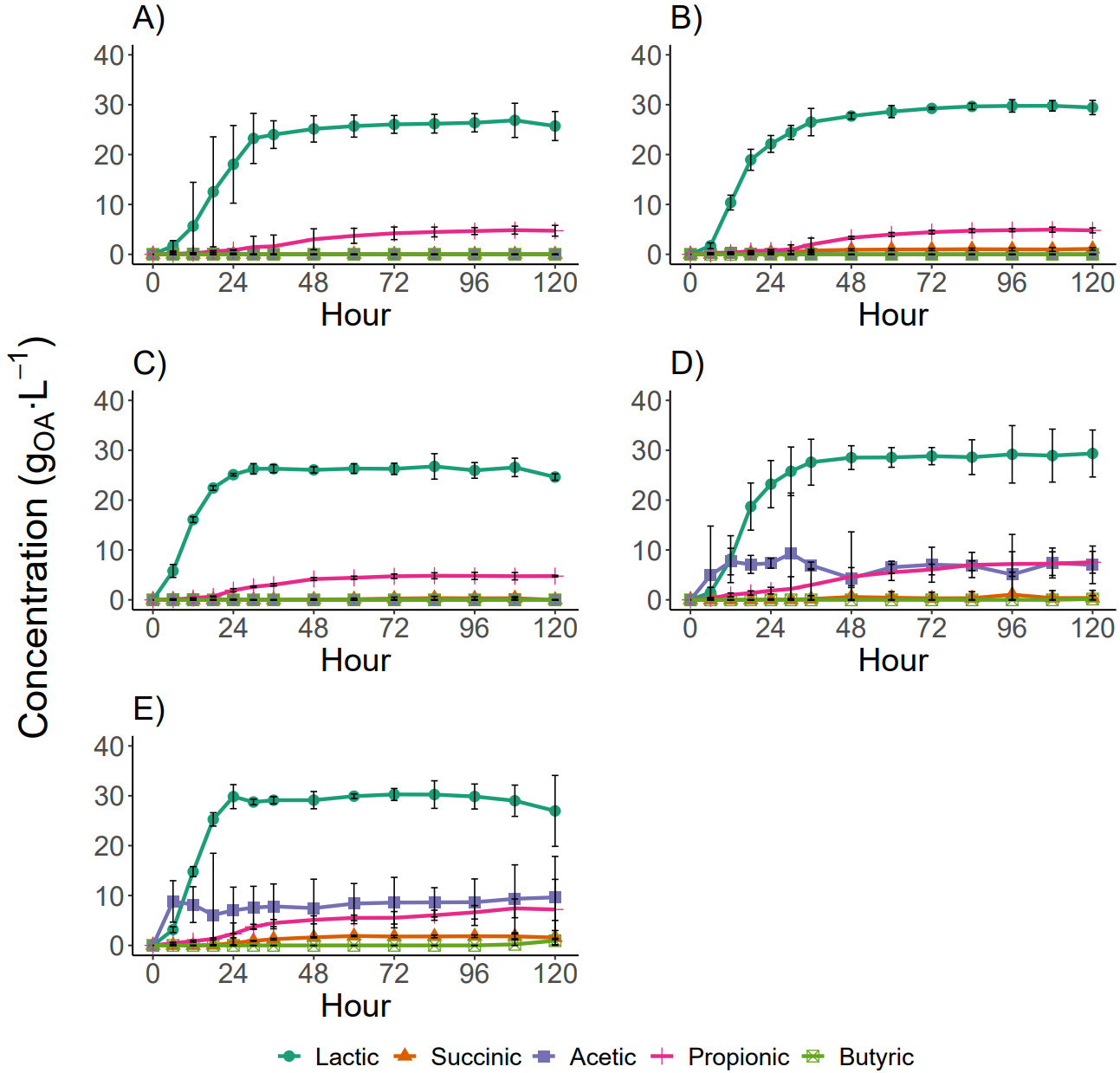


**Figure S3:** Plot of organic acid production with 0 g·L^-1^ sucrose addition with A) 0 mg_N_·L^-1^ supplement, B) 300 mg_N_·L^-1^ supplement with NH_4_Cl, C) 400 mg_N_·L^-1^ supplement with NH_4_Cl, D) 300 mg_N_·L^-1^ supplement with digestate, and E) 400 mg_N_·L^-1^ supplement with digestate. Error bars represent the mean ±95% confidence interval.


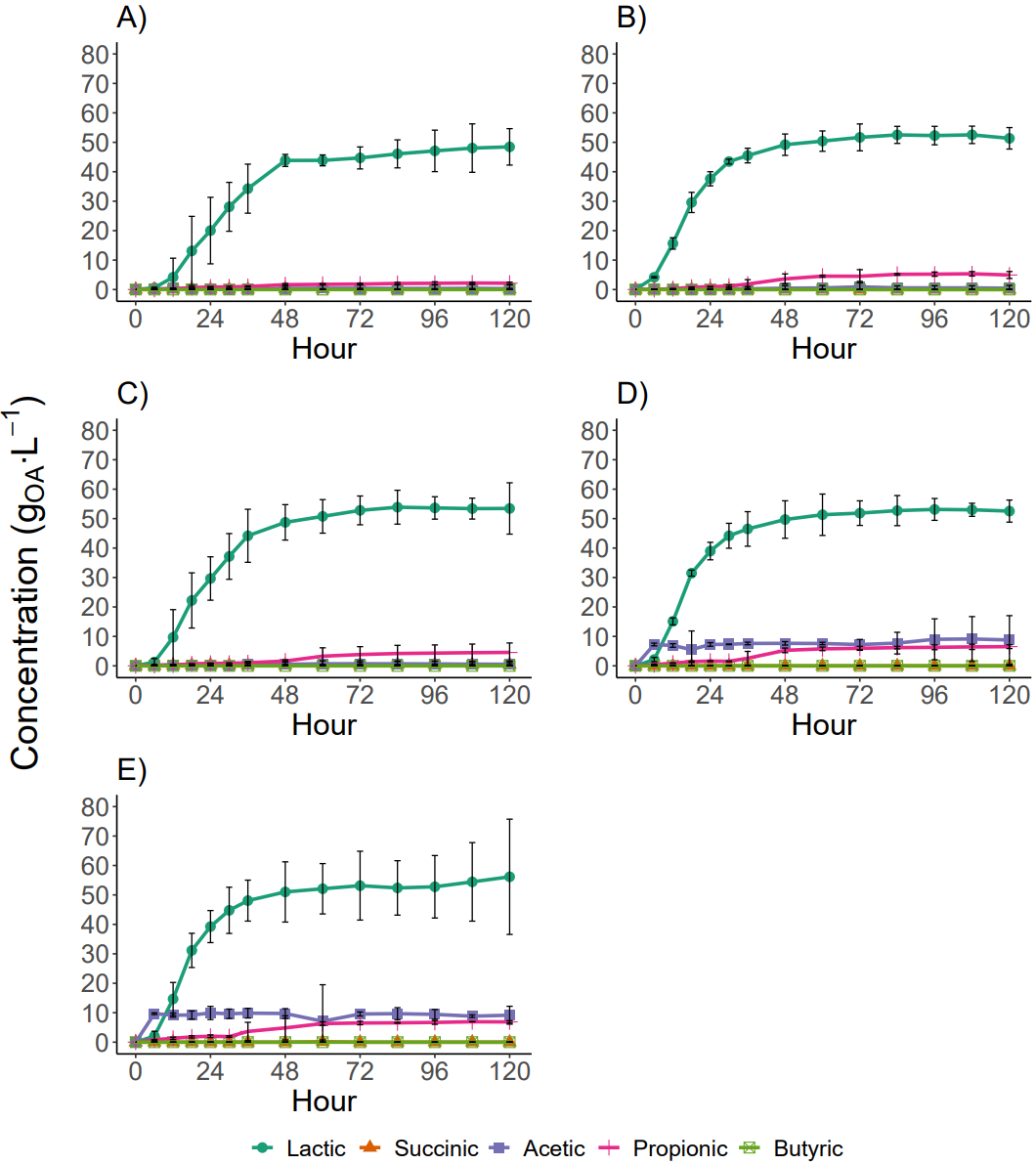


**Figure S4:** Plot of organic acid production with 43 g·L^-1^ sucrose addition with A) 0 mg_N_·L^-1^ supplement, B) 300 mg_N_·L^-1^ supplement with NH_4_Cl, C) 400 mg_N_·L^-1^ supplement with NH_4_Cl, D) 300 mg_N_·L^-1^ supplement with digestate, and E) 400 mg_N_·L^-1^ supplement with digestate. Error bars represent the mean ±95% confidence interval.


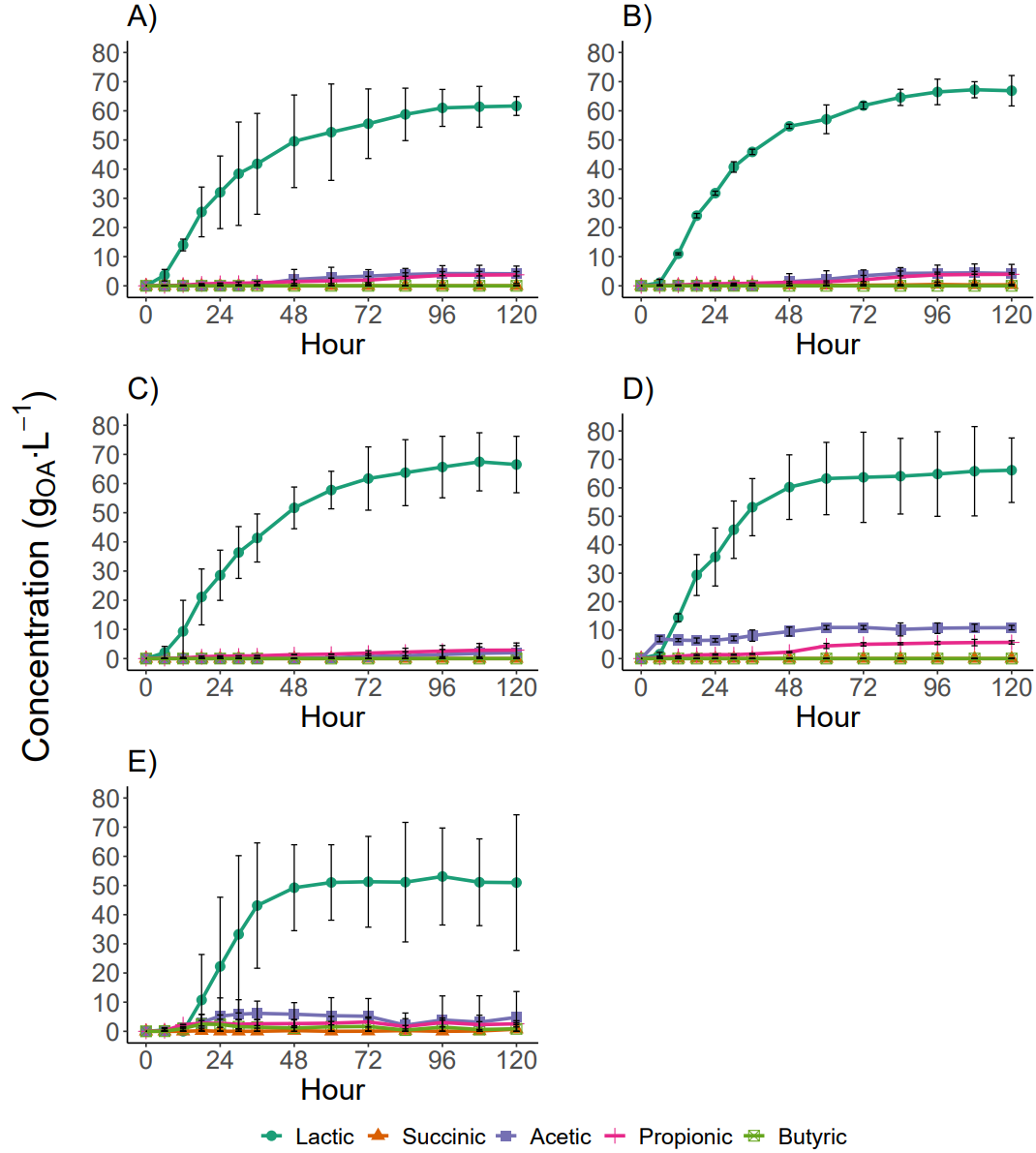


**Figure S5:** Plot of organic acid production with 107 g·L^-1^ sucrose addition with A) 0 mg_N_·L^-1^ supplement, B) 300 mg_N_·L^-1^ supplement with NH_4_Cl, C) 400 mg_N_·L^-1^ supplement with NH_4_Cl, D) 300 mg_N_·L^-1^ supplement with digestate, and E) 400 mg_N_·L^-1^ supplement with digestate. Error bars represent the mean ±95% confidence interval.


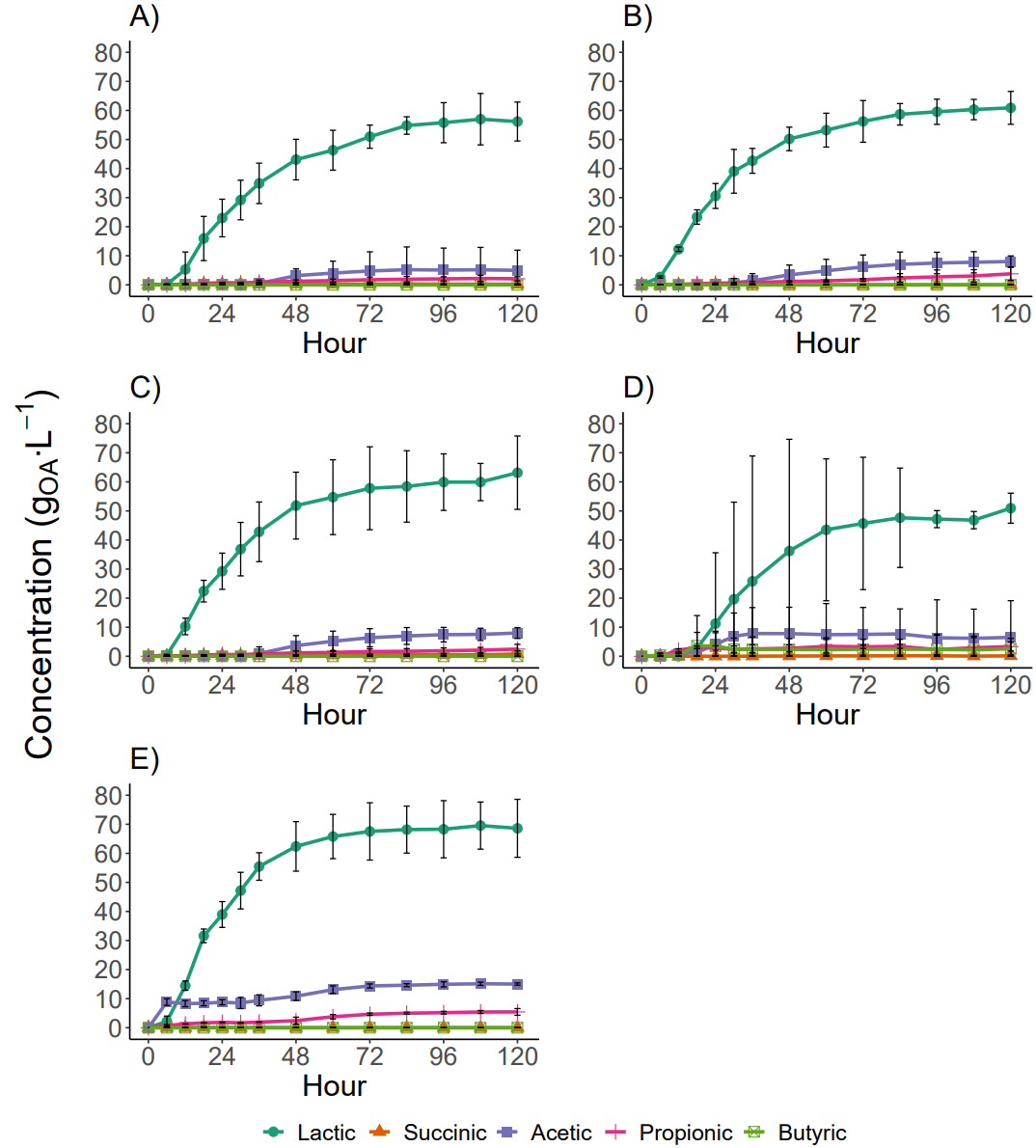


**Figure S6:** Plot of organic acid production with 150 g·L^-1^ sucrose addition with A) 0 mg_N_·L^-1^ supplement, B) 300 mg_N_·L^-1^ supplement with NH_4_Cl, C) 400 mg_N_·L^-1^ supplement with NH_4_Cl, D) 300 mg_N_·L^-1^ supplement with digestate, and E) 400 mg_N_·L^-1^ supplement with digestate. Error bars represent the mean ±95% confidence interval.


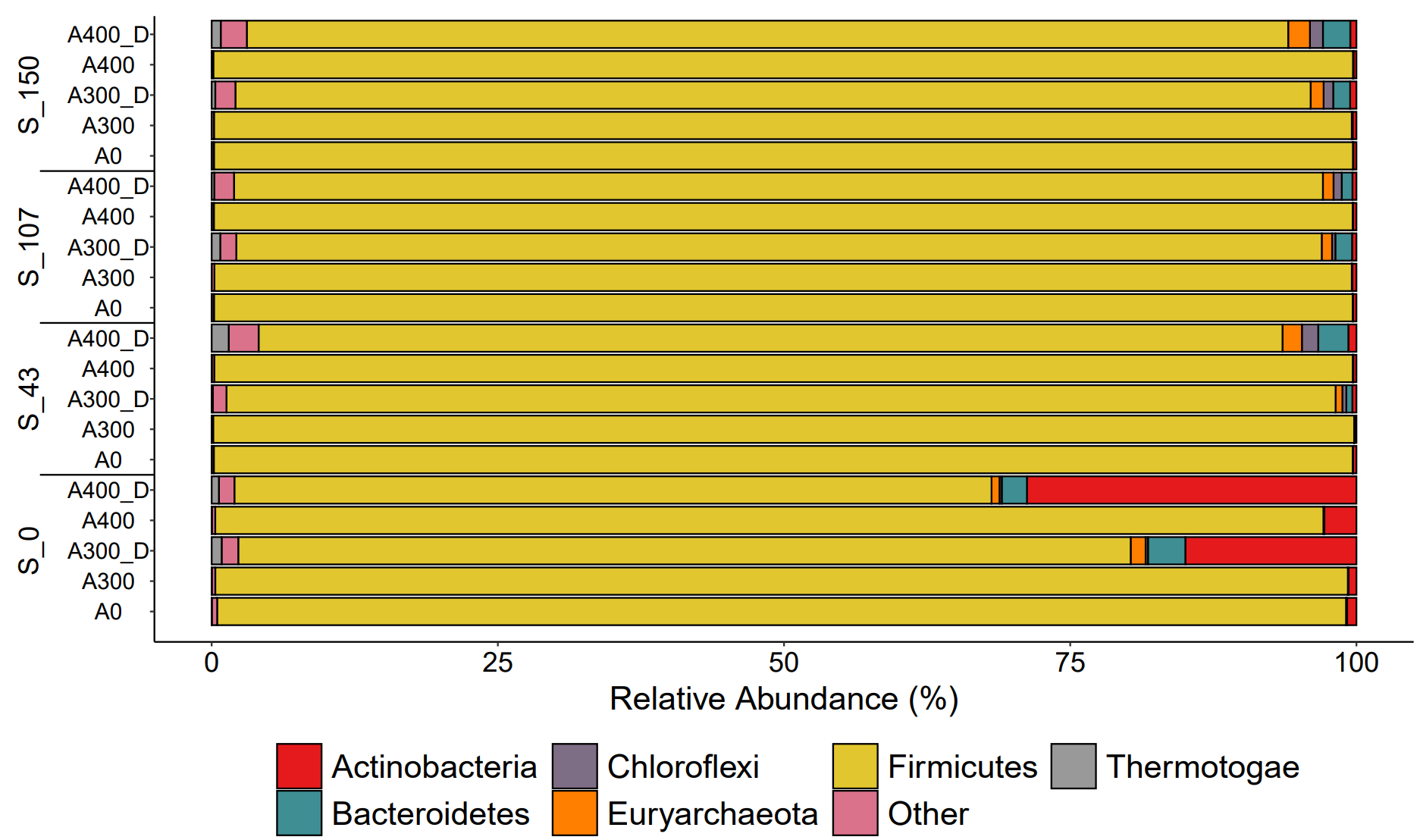


**Figure S7:** Relative abundance of microbial phyla (>1%) following 5-days of FW fermentation supplemented with sucrose and N (NH4Cl or digestate). Sucrose level is denoted by an “S” followed by the initial supplement concentration, while supplemented N is denoted by an “A” and N added as digestate is denoted by “D”.


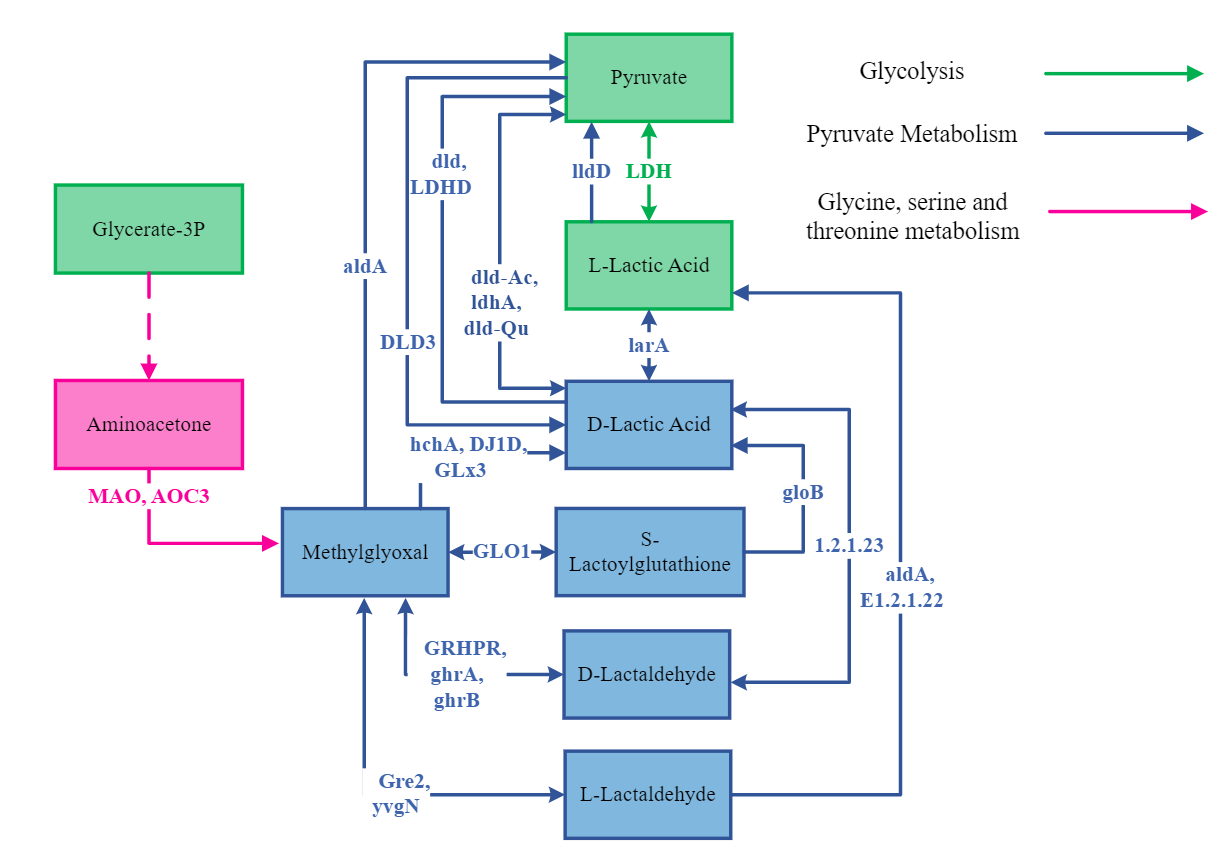


**Figure S8:** Identified genes within selected metabolic pathways associated with LA production and consumption as well as other organic acids. All genes were identified via the KEGG database. To simplify the diagram, only pathways directly related to LA formation have been included (Modified from (KEGG, 2022)).


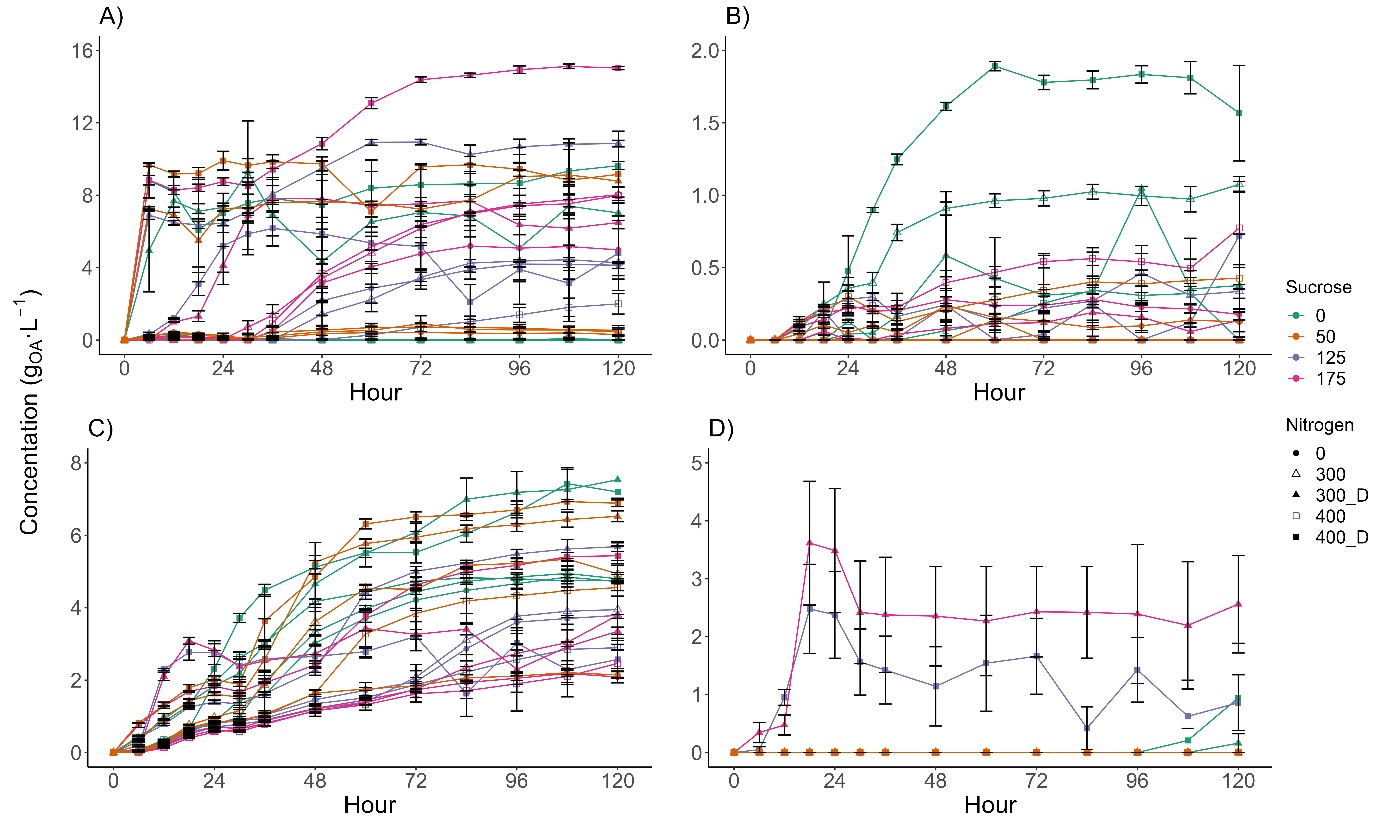


**Figure S9:** Production curves of A) Acetic acid, B) Succinic Acid, C) Propionic Acid, and D) Butyric Acid. Values are presented as the mean of triplicates. Error bars represent the mean ± the standard error.

**Tables**

**Table S1:** Synthetic food waste composition (Capson-Tojo et al., 2017)

| Ingredient | Proportion (% wet basis) |
| --- | --- |
| Apples | 25.9 |
| Lettuce | 25.9 |
| Potato | 25.9 |
| Pasta (Cooked) | 1.2 |
| Rice (Cooked) | 1.2 |
| Flour | 1.2 |
| Cereals | 1.2 |
| Bread | 6.2 |
| Chicken | 4.1 |
| Beef | 4.1 |
| Cheese | 1.9 |
| Biscuits | 1.5 |

**Table S2:** Composition of the synthetic food waste, inoculum, and digestate utilised within this study. Analyses were conducted by ARL (Welshpool, Western Australia)

|  | Synthetic Food waste | Inoculum | Digestate | HCC ^a^ |
| --- | --- | --- | --- | --- |
| Potassium - Total (mg/L) | 870 | 840 | 900 | 871.79 |
| Magnesium - Total (mg/L) | 59 | 130 | 110 | 72.48 |
| Calcium - Total (mg/L) | 140 | 1,500 | 1,600 | 468.38 |
| Sodium - Total (mg/L) | 280 | 940 | 1,000 | 441.03 |
| Sulphur - Total (mg/L) | 150 | 240 | 120 | 153.33 |
| Iron - Total (mg/L) | 2.3 | 110 | 93 | 24.68 |
| Manganese - Total (mg/L) | 1.5 | 5.1 | 5 | 2.32 |
| Copper - Total (mg/L) | 0.38 | 1.3 | 1.4 | 0.61 |
| Cobalt - Total (mg/L) | 0.005 | 0.082 | 0.071 | 0.02 |
| Zinc - Total (mg/L) | 2.9 | 9.3 | 10 | 4.48 |
| Nickel - Total (mg/L) | 0.02 | 0.22 | 0.17 | 0.06 |
| Total Nitrogen (mg/L) | 3,000 | 3,000 | 3,400 | 3,058.12 |
| Total Phosphorus (mg/L) | 110 | 290 | 370 | 163.16 |
| Chloride (mg/L) | 1,100 | 1,800 | 1,900 | 1,276.07 |
| Ammonia-N (mg/L) | 130 | 570 | 2,500 | 511.97 |
| NOx-N (mg/L) | <1 | <1 | 14 | 2.03 |
| Nitrate-N (mg/L) | <1 | <1 | 14 | 2.03 |

a: Highest Calculated combined Concentration (HCC) during lactic acid fermentation (i.e. with 400 mg_N_·L^-1^ as digestate)

**Table S3:** Factorial design for the FW fermentation experiments with sucrose and digestate supplementation.

| Block* | Sucrose | NH_4_Cl | Digestate | Linearised Sucrose | Linearised N dosage |
| --- | --- | --- | --- | --- | --- |
| 1 | 0 | 0 | 0 | 0.00 | 0.00 |
| 1 | 107 | 0 | 0 | 0.71 | 0.00 |
| 1 | 43 | 300 | 0 | 0.29 | 0.75 |
| 1 | 150 | 300 | 0 | 1.00 | 0.75 |
| 1 | 0 | 400 | 0 | 0.00 | 1.00 |
| 1 | 107 | 400 | 0 | 0.71 | 1.00 |
| 2 | 43 | 0 | 0 | 0.29 | 0.00 |
| 2 | 150 | 0 | 0 | 1.00 | 0.00 |
| 2 | 0 | 300 | 0 | 0.00 | 0.75 |
| 2 | 107 | 300 | 0 | 0.71 | 0.75 |
| 2 | 3 | 400 | 0 | 0.02 | 1.00 |
| 2 | 150 | 400 | 0 | 1.00 | 1.00 |
| 3 | 0 | 0 | 300 | 0.00 | 0.75 |
| 3 | 10 | 0 | 300 | 0.07 | 0.75 |
| 3 | 107 | 0 | 300 | 0.71 | 0.75 |
| 3 | 0 | 0 | 400 | 0.00 | 1.00 |
| 3 | 43 | 0 | 400 | 0.29 | 1.00 |
| 3 | 150 | 0 | 400 | 1.00 | 1.00 |
| 4 | 150 | 0 | 300 | 1.00 | 0.75 |
| 4 | 107 | 0 | 400 | 0.71 | 1.00 |
| *Blocks 1-4 were performed in time sequence | | | |  |  |

**Table S4:** Organic acid concentration (g·L^-1^) RSM model output and selectivity, provided with (±)95% confidence intervals.

| Block | Sucrose | N | Source^a^ | Lactic | Succinic | Acetic | Propionic | Butyric | Selectivity (gLA_COD_/TCOD)^b^ |
| --- | --- | --- | --- | --- | --- | --- | --- | --- | --- |
| 1 | 0 | 0 | 0 | 27.34(±3.44) | 0.18(±0.26) | 1.08(±1.63) | 3.37(±0.89) | -0.22(±0.37) | 83.0% |
| 2 | 0 | 300 | 0 | 26.28(±3.19) | 0.7(±0.23) | -0.71(±1.4) | 4.14(±0.74) | 0.3(±0.4) | 80.7% |
| 3 | 0 | 300 | 1 | 27.29(±2.84) | 0.88(±0.27) | 5.35(±1.58) | 5.17(±0.65) | -0.12(±0.42) | 67.4% |
| 1 | 0 | 400 | 0 | 32.55(±3.1) | 0.38(±0.24) | 1.48(±1.42) | 4.39(±0.72) | -0.22(±0.37) | 81.0% |
| 3 | 0 | 400 | 1 | 28.59(±2.84) | 1.21(±0.27) | 6.87(±1.58) | 5.92(±0.7) | -0.12(±0.42) | 64.0% |
| 2 | 50 | 0 | 0 | 44.44(±3.36) | 0.15(±0.24) | -0.5(±1.71) | 2.5(±0.71) | 0.28(±0.3) | 92.4% |
| 1 | 50 | 300 | 0 | 53.32(±2.48) | -0.07(±0.18) | 1.89(±1.17) | 5.24(±0.63) | -0.24(±0.29) | 85.8% |
| 3 | 50 | 300 | 1 | 49.36(±2.48) | 0.18(±0.22) | 5.86(±1.53) | 5.14(±0.6) | 0.16(±0.29) | 78.5% |
| 2 | 50 | 400 | 0 | 49.65(±3.02) | 0.35(±0.21) | -0.11(±1.52) | 3.14(±0.67) | 0.28(±0.3) | 90.7% |
| 3 | 50 | 400 | 1 | 50.67(±2.48) | 0.51(±0.22) | 7.38(±1.53) | 5.79(±0.61) | 0.16(±0.29) | 75.7% |
| 1 | 125 | 0 | 0 | 62.96(±3.36) | -0.3(±0.24) | 4.25(±1.6) | 3.54(±0.71) | -0.28(±0.3) | 88.1% |
| 2 | 125 | 300 | 0 | 61.89(±2.48) | 0.22(±0.18) | 2.45(±1.12) | 3.58(±0.63) | 0.24(±0.29) | 88.4% |
| 3 | 125 | 300 | 1 | 62.91(±2.59) | -0.34(±0.23) | 8.52(±1.5) | 4.61(±0.61) | 0.59(±0.32) | 80.0% |
| 1 | 125 | 400 | 0 | 68.17(±3.02) | -0.09(±0.21) | 4.65(±1.39) | 3.59(±0.67) | -0.28(±0.3) | 88.2% |
| 4 | 125 | 400 | 1 | 59.24(±3.04) | 0.36(±0.25) | 7.94(±1.76) | 3.99(±0.7) | 1.11(±0.36) | 79.0% |
| 2 | 175 | 0 | 0 | 53.98(±3.44) | 0.37(±0.26) | 5.2(±1.76) | 2.02(±0.89) | 0.22(±0.37) | 86.1% |
| 1 | 175 | 300 | 0 | 62.86(±3.19) | 0.15(±0.23) | 7.59(±1.63) | 4.02(±0.74) | -0.3(±0.4) | 83.0% |
| 4 | 175 | 300 | 1 | 53.93(±3.23) | 0.02(±0.28) | 9.46(±1.9) | 2.8(±0.72) | 1.4(±0.44) | 77.3% |
| 2 | 175 | 400 | 0 | 59.19(±3.1) | 0.57(±0.24) | 5.6(±1.57) | 1.68(±0.72) | 0.22(±0.37) | 87.0% |
| 3 | 175 | 400 | 1 | 60.2(±3.05) | -0.01(±0.29) | 13.08(±1.84) | 4.34(±0.73) | 0.87(±0.46) | 74.4% |

a. Nitrogen source, 0 = NH_4_Cl, 1 = Digestate, b. calculated based on measured VFAs.

**Table S5:** Microbial community RSM model parameters with (±)95% confidence intervals.

| Genus | Intercept | R_B_ | β_S_ | β_N_ | β_NS_ | β_S2_ | β_N 2_ | β_S_N_ | β_S_NS_ | β_N_NS_ | Adj.R^2^ |
| --- | --- | --- | --- | --- | --- | --- | --- | --- | --- | --- | --- |
| Allisonella | 0.13 (±0.21) | - | -1.28 (±0.73) *** | 0.00 (±0.21) | 1.75 (±0.82) *** | 1.28 (±0.69) *** | - | - | -1.11 (±0.37) *** | -0.99 (±0.92) * | 0.61 |
| Bacillales  unclassified | 1.97 (±0.42) *** | - | -3.67 (±1.28) *** | -1.58 (±0.55) *** | -0.41 (±0.41) | 1.5 (±1.11) ** | - | 1.89 (±0.87) *** | 0.50 (±0.65) | - | 0.56 |
| Bifidobacterium | 2.33 (±3.34) | - | -21.01 (±11.77) *** | 0.42 (±3.45) | 3.72 (±13.3) | 20.41 (±11.14) *** | - | - | -16.12 (±6.03) *** | 10.29 (±14.79) | 0.57 |
| Clostridiaceae 1 unclassified | -0.40 (±0.44) | 0.32 (±0.23) ** | -0.01 (±0.35) | 0.07 (±0.32) | -0.75 (±1.27) | - | - | - | -0.58 (±0.56) * | 1.00 (±1.36) | 0.29 |
| CSS_15 | 7.20 (±5.58) * | -1.93 (±2.67) | -29.95 (±13.45) *** | 15.83 (±5.78) *** | -9.10 (±5.84) ** | 28.47 (±11.65) *** | - | -20.17 (±9.19) *** | 16.07 (±7.00) *** | - | 0.64 |
| CSS_18 | 0.64 (±1.37) | - | -6.24 (±4.84) * | 0.00 (±1.42) | 11.01 (±5.47) *** | 6.24 (±4.58) ** | - | - | -5.37 (±2.48) *** | -7.56 (±6.08) * | 0.47 |
| CSS_7 | 0.61 (±1.79) | - | -5.73 (±6.30) | 0.00 (±1.85) | -4.22 (±7.12) | 5.73 (±5.96) | - | - | -5.02 (±3.23) ** | 10.25 (±7.91) * | 0.37 |
| Defluviitoga | -0.31 (±0.31) * | 0.21 (±0.17) * | - | 0.00 (±0.24) | -0.68 (±0.95) | - | - | - |  | 1.10 (±1.04) * | 0.54 |
| DTU014_ge | -0.06 (±0.12) | - | 0.61 (±0.44) ** | 0.00 (±0.13) | -0.35 (±0.49) | -0.61 (±0.41) ** | - | - | 0.23 (±0.22) * | 0.99 (±0.55) *** | 0.81 |
| Lactobacillaceae  unclassified | 0.75 (±0.61) * | 0.32 (±0.33) | - | -2.69 (±1.77) ** | -0.72 (±0.68) * | - | 1.78 (±1.73) * | - | - | - | 0.33 |
| Lactobacillales  unclassified | 1.17 (±0.23) *** | -0.21 (±0.12) ** | -1.55 (±0.55) *** | -0.05 (±0.16) | -0.08 (±0.65) | 1.11 (±0.52) *** | - | - | 0.25 (±0.29) | 0.80 (±0.69) * | 0.66 |
| Lactobacillus | 77.44 (±11.78) *** | -3.93 (±5.58) | 77.58 (±28.16) *** | 31.01 (±29.71) * | -15.37 (±12.24) * | -66.97 (±24.38) *** | -39.65 (±27.5) ** | 18.82 (±19.23) | 14.91 (±14.66) * | - | 0.72 |
| Leuconostoc | 3.83 (±0.62) *** | - | -5.75 (±2.23) *** | 0.61 (±0.63) | -1.79 (±0.74) *** | 2.89 (±2.11) ** | - | - | 2.37 (±1.14) *** |  | 0.56 |
| Olsenella | 0.10 (±0.28) | - | -0.93 (±0.99) | 0 (±0.29) | -1.00 (±1.12) | 0.93 (±0.94) | - | - | -0.79 (±0.51) ** | 1.87 (±1.25) ** | 0.33 |
| Pediococcus | 4.23 (±5.28) | 3.3 (±2.88) * | - | -20.97 (±15.41) ** | -6.53 (±5.88) * | - | 12.79 (±15.08) | - | - | - | 0.80 |
| Proteiniphilum | -0.31 (±0.44) | 0.29 (±0.25) * | -0.99 (±1.15) | - | 1.69 (±0.53) *** | 0.94 (±1.09) | - | - | -1.63 (±0.60) *** | - | 0.32 |
| Streptococcus | 0.28 (±1.1) | - | -2.68 (±3.89) | 0.00 (±1.14) | -1.00 (±4.4) | 2.67 (±3.68) | - | - | -3.26 (±1.99) ** | 5.24 (±4.89) * | 0.77 |
| Syntrophaceticus | 0.00 (±0.18) | - | 0.60 (±0.66) | -0.01 (±0.20) | -0.07 (±0.75) | -0.60 (±0.64) | - | - | - | 1.15 (±0.85) ** | 0.43 |

***=(P<0.001), **=(P<0.01), *=(P<0.05).

**Table S6:** Functional gene RSM model parameters with (±) 95% confidence intervals. Note that the relative abundance of genes were scaled by 1,000 (Section 2.6 in MS).

| Gene | Intercept | R_B_ | β_S_ | β_N_ | β_NS_ | β_S2_ | β_N 2_ | β_S_N_ | β_S_NS_ | β_N_NS_ | Adj.R^2^ |
| --- | --- | --- | --- | --- | --- | --- | --- | --- | --- | --- | --- |
| aldA | 5.22 (±1.08)³ | - | -8.48  (±3.51)³ | 2.17  (±1.32)² | -1.06  (±1.31) | 4.69  (±3.15)² | - | -1.64  (±1.81) | 3.61  (±1.70)³ | -1.51  (±1.39)¹ | 0.62 |
| AOC3 | 2.64 (±0.54)³ | - | -4.34  (±1.74)³ | 1.08  (±0.66)² | -0.59  (±0.65) | 2.42  (±1.56)² | - | -0.80  (±0.90) | 1.84  (±0.84)³ | -0.75  (±0.69)¹ | 0.62 |
| dld_Qu | 2.64 (±0.53)³ | - | -4.36  (±1.72)³ | 1.07  (±0.65)² | -0.63  (±0.64) | 2.45  (±1.55)² | - | -0.80  (±0.89) | 1.83  (±0.83)³ | -0.77  (±0.68)¹ | 0.63 |
| E2.3.1.8 | -0.08  (±0.46) | 0.24 (±0.24)¹ | -0.95  (±1.12) | -0.01  (±0.32) | 0.51  (±0.57) | 0.76  (±1.05) | - | - | -0.69  (±0.58)¹ | 0.39  (±0.47) | 0.54 |
| fucO | 0.09  (±0.21) | - | -0.67  (±0.95) | - | 0.44  (±0.30)² | 0.66  (±0.90) | - | - | -0.54  (±0.48)¹ | - | 0.15 |
| ghrA | 5.59 (±1.10)³ | - | -9.44  (±3.57)³ | 2.18  (±1.35)² | -1.10  (±1.33) | 5.43  (±3.20)² | - | -1.69  (±1.84) | 3.72  (±1.73)³ | -1.50  (±1.41)¹ | 0.63 |
| ghrB | 8.47 (±1.70)³ | - | -16.45  (±6.07)³ | 0.75  (±1.75) | -3.84  (±2.37)² | 10.33  (±5.71)³ | - | - | 7.17  (±3.07)³ | -2.12  (±2.52) | 0.49 |
| gldA | 22.81 (±5.23)³ | 8.43  (±3.07)³ | -7.53  (±3.80)³ | -23.22 (±20.48)¹ | -10.88  (±6.22)³ | - | 20.19  (±20.87) | - | - | - | 0.48 |
| GLO1 | 5.57 (±1.15)³ | - | -9.61  (±4.22)³ | 2.53  (±1.41)³ | 1.99  (±0.80)³ | 6.19  (±3.88)² | - | -2.53  (±2.23)¹ | - | - | 0.69 |
| gloB | 43.19 (±10.45)³ | 16.87 (±6.13)³ | -13.08  (±7.60)² | -46.86 (±40.90)¹ | -21.75  (±12.42)³ | - | 40.36  (±41.69) | - | - | - | 0.48 |
| hchA | 0.00 (±0.01)¹ | 0.00  (±0.01)³ | 0.00  (±0.01)² | - | - | 0.00  (±0.01)² | - | - | - | - | 0.23 |
| K15024 | 0.00  (±0.72) | - | 0.00  (±0.87) | 0.00  (±0.79) | 1.73  (±1.07)² | - | - | - | -2.03  (±1.38)² | 0.79  (±1.14) | 0.34 |
| LDH | 0.05  (±0.49) | - | -0.03  (±0.60) | 0.01  (±0.54) | 1.39  (±0.73)³ | - | - | - | -1.00  (±0.95)¹ | 0.59  (±0.78) | 0.46 |
| ldhA | 2.66  (±0.60)³ | - | -4.20  (±1.97)³ | 1.05  (±0.74)² | -0.13  (±0.73) | 2.24  (±1.77)¹ | - | -0.74  (±1.02) | 1.74  (±0.95)³ | -0.67  (±0.78) | 0.57 |
| MAO | 63.39 (±6.33)³ | -6.8  (±3.26)³ | 23.01 (±16.03)² | 18.34  (±21.53) | 12.00  (±7.12)² | -11.75  (±14.35) | -19.82  (±21.52) | 6.88  (±8.36) | -8.20  (±7.85)¹ | - | 0.55 |
| pct | 0.01  (±0.28) | - | 0.00  (±0.44) | - | 0.97  (±0.44)³ | - | - | - | -1.03  (±0.70)² | - | 0.28 |
| pta | 24.95 (±5.09)³ | -7.95  (±2.99)³ | 7.19  (±3.7)³ | 22.85 (±19.92)¹ | 9.96  (±6.05)² | - | -19.83  (±20.31) | - | - | - | 0.48 |
| yqhD | 22.84 (±5.22)³ | 8.47  (±3.06)³ | -7.40  (±3.79)³ | -23.36 (±20.41)¹ | -11.44  (±6.20)³ | - | 20.21  (±20.81) | - | - | - | 0.48 |

3=(P<0.001), 2=(P<0.01), 1=(P<0.05).
